# *In silico* Design of Phl p 6 Variants With Altered Fold-Stability Significantly Impacts Antigen Processing, Immunogenicity and Immune Polarization

**DOI:** 10.3389/fimmu.2020.01824

**Published:** 2020-08-18

**Authors:** Petra Winter, Stefan Stubenvoll, Sandra Scheiblhofer, Isabella A. Joubert, Lisa Strasser, Carolin Briganser, Wai Tuck Soh, Florian Hofer, Anna Sophia Kamenik, Valentin Dietrich, Sara Michelini, Josef Laimer, Peter Lackner, Jutta Horejs-Hoeck, Martin Tollinger, Klaus R. Liedl, Johann Brandstetter, Christian G. Huber, Richard Weiss

**Affiliations:** ^1^Department of Biosciences, University of Salzburg, Salzburg, Austria; ^2^Center of Molecular Biosciences & Institute of General, Inorganic and Theoretical Chemistry, University of Innsbruck, Innsbruck, Austria; ^3^Center of Molecular Biosciences & Institute of Organic Chemistry, University of Innsbruck, Innsbruck, Austria

**Keywords:** structural stability, endolysosomal degradation, antigen processing and presentation, protein stabilization, immune polarization, *in silico* mutagenesis, *in silico* mutant screening

## Abstract

**Introduction:** Understanding, which factors determine the immunogenicity and immune polarizing properties of proteins, is an important prerequisite for designing better vaccines and immunotherapeutics. While extrinsic immune modulatory factors such as pathogen associated molecular patterns are well-understood, far less is known about the contribution of protein inherent features. Protein fold-stability represents such an intrinsic feature contributing to immunogenicity and immune polarization by influencing the amount of peptide-MHC II complexes (pMHCII). Here, we investigated how modulation of the fold-stability of the grass pollen allergen Phl p 6 affects its ability to stimulate immune responses and T cell polarization.

**Methods:** MAESTRO software was used for *in silico* prediction of stabilizing or destabilizing point mutations. Mutated proteins were expressed in *E. coli*, and their thermal stability and resistance to endolysosomal proteases was determined. Resulting peptides were analyzed by mass spectrometry. The structure of the most stable mutant protein was assessed by X-ray crystallography. We evaluated the capacity of the mutants to stimulate T cell proliferation *in vitro*, as well as antibody responses and T cell polarization *in vivo* in an adjuvant-free BALB/c mouse model.

**Results:** In comparison to wild-type protein, stabilized or destabilized mutants displayed changes in thermal stability ranging from −5 to +14°. While highly stabilized mutants were degraded very slowly, destabilization led to faster proteolytic processing *in vitro*. This was confirmed in BMDCs, which processed and presented the immunodominant epitope from a destabilized mutant more efficiently compared to a highly stable mutant. *In vivo*, stabilization resulted in a shift in immune polarization from TH2 to TH1/TH17 as indicated by higher levels of IgG2a and increased secretion of TNF-α, IFN-γ, IL-17, and IL-21.

**Conclusion:** MAESTRO software was very efficient in detecting single point mutations that increase or reduce fold-stability. Thermal stability correlated well with the speed of proteolytic degradation and presentation of peptides on the surface of dendritic cells *in vitro*. This change in processing kinetics significantly influenced the polarization of T cell responses *in vivo*. Modulating the fold-stability of proteins thus has the potential to optimize and polarize immune responses, which opens the door to more efficient design of molecular vaccines.

## Introduction

An important question for the design and understanding of molecular vaccines is how protein intrinsic factors determine the immunogenic properties of an antigen. Several parameters have been identified that can have a strong impact on the immunogenicity and immune polarizing potential of allergens, like aggregation behavior (1), glycosylation (2), molecular mimicry (3), or enzymatic activity (4). However, one of the most important underlying principles is that for a strong immune response sufficient amounts of peptides have to be presented on major histocompatibility complex molecules to provide optimal stimulation for T cells (5). Moreover, the amount of peptide-MHC complexes (pMHC) on the surface of antigen presenting cells (APCs) can influence the polarization of naïve T helper (TH) cells. While in the classic qualitative model, TH cell polarization is mainly determined by cytokines secreted by APCs, there is growing evidence for an important role of strength and duration of the pMHCII—T cell receptor (TCR) interaction. In favor of the quantitative model, it has been consistently shown *in vitro* that low antigen doses promote TH2 polarization, while high antigen doses induce IFN-γ secreting TH1 cells (6, 7). More recently, van Panhuys et al. have shown for the first time *in vivo* that the quantity of antigen presented by a dendritic cell (DC) may overrule qualitative signals provided by the same DC, thus shifting T cell polarization from either TH1 to TH2 or vice versa (8). Consequently, a model emerges, where depending on the pMHCII—TCR interaction, a DC can induce no response, anergy, TH2 polarization, TH1 polarization, or activation induced cell death with increasing signal strength (9). Moreover, it has been shown that TCR signaling strength is also crucial for the induction of Tfh polarization (10–12), persistence of Foxp3^+^ Tregs (13, 14), and differentiation of TH17 effector cells (15). These findings have important implications for the design of novel vaccines, and for our understanding why some proteins are potent TH2 inducers (allergens), while other proteins induce TH1 responses [e.g., viral proteins (16)].

Based on this concept, the overall fold-stability of an antigen has been suggested as an important protein intrinsic parameter that can influence immunogenicity and immune polarization. Proteins with a high conformational stability are on the one hand more resistant to proteases, which are abundant on skin, mucosal surfaces and in the extracellular matrix (17, 18), and on the other hand also frequently display enhanced resistance against proteolytic digestion in the endolysosomal compartment of APCs. Hence, fold-stability substantially controls the cell surface density of pMHCII molecules specific for a given antigen, thereby influencing the immune polarization of T cells. Moreover, hyper-stable proteins, which resist proteolysis within the antigen processing compartment, may escape into the cytoplasm of APCs and enter the cross—presentation pathway through the proteasome. The resulting peptides finally end up on MHC I and are presented to CD8 T cells. Several studies supporting these ideas were reviewed by Scheiblhofer et al. (19). However, the results described are inconsistent, as different methods were used to modulate protein stability and different experimental settings were applied.

Various methods have been used to manipulate the conformational stability of a protein such as the introduction of cysteine bonds (20, 21), pairing of charges (22), or chemical cross linking (23, 24). However, the outcome of such mutations and the immunological effects of chemical cross linkers are often difficult to predict. In our current work we therefore employed an *in silico* mutagenesis approach using MAESTRO (25), a software tool, which predicts the effect of point mutations on the free energy (ΔΔG) of the molecule. We used the grass pollen allergen Phl p 6, which is a small globular protein of 11.8 kD that can be easily expressed in *E. coli* and is a known inducer of TH2 responses. We selected single point mutations that either stabilized or destabilized Phl p 6 without changing immunodominant T cell epitopes or the overall structure of the protein. Using these mutant proteins, we investigated the effect of fold-stability on antigen processing and presentation, as well as on immunogenicity and immune polarization *in vitro* and *in vivo*.

## Materials and Methods

### *In silico* Mutagenesis

Stabilizing or destabilizing mutations were selected *in silico* using the MAESTRO software tool (25) for predicting the influence of single point mutations or combinations thereof on protein stability. The change in stability predictions are based on the PDB entry 1NLX chain L. The structurally unresolved residues at the N- and C-terminus were modeled using the UCSF Chimera (26) interface to MODELER (27). The final model was subjected to a MAESTRO greedy search, excluding the major T cell epitopes 65–79 (DEVYNAAYNAADHAA) and 92–106 (SEALRIIAGTPEVHA). Mutations with a relative accessible surface area or of <30% were defined as buried residues and >30% as surface exposed residues.

### Expression and Purification of Recombinant Proteins

Wild-type Phl p 6 and its mutants were expressed from pET17b constructs in *Escherichia coli* strain BL21 Star (DE3; Invitrogen/Thermo Fisher Scientific). Cells were grown in auto-inducing ZYM 5052 medium (28) supplemented with 100 mg/L ampicillin in baffled flasks at 37°C for 20 h. Cells were lysed in 6 mL lysis buffer (25 mM imidazole, 0.5 mM EDTA, pH 7.4) per gram pellet weight and 300 μg/mL lysozyme was added. After stirring for 30 min at RT, bacterial cells were lysed by three freeze/thaw cycles at 70°C. DNA was digested by addition of 100 μg DNAse I (Roche) per gram pellet weight and stirring at RT for 60 min. After centrifugation (20,000 g, 45 min, 5°C), unwanted proteins were removed from the supernatant by 30% ammonium sulfate precipitation. The supernatant was further purified using hydrophobic interaction chromatography (Phenylsepharose 6, fast flow, GE Healthcare) using 25 mM imidazole, 1.25 M ammonium sulfate, pH 7.5 as binding buffer and 25 mM imidazole pH 7.4 for the elution gradient. Fractions were analyzed on a 15% acrylamide gel by SDS PAGE and Phl p 6 containing fractions were pooled and dialyzed three times against 25 mM imidazole, 4% 2-propanol, pH 7.4. Dialyzed pooled fractions were applied to anion exchange chromatography (DEAE sepharose fast flow, GE Healthcare) using 20 mM imidazole, 4% 2-propanol, pH 7.4 for equilibration and washing. Elution was performed with a NaCl gradient in the same buffer. Fractions containing Phl p 6 were finally purified by size exclusion chromatography using a 100/60 sephacryl S200HR (GE Healthcare) column equilibrated with 2 mM sodium phosphate buffer, pH 7.4.

^15^N and ^15^N/^13^C labeled protein samples for NMR spectroscopy were recombinantly produced using M9 minimal media supplemented with ^15^NH_4_Cl and ^13^C-D-glucose and purified as described above.

Endotoxin content was determined by *Limulus* amebocyte assay (PYROTELL^®^-T, Associates of Cape Cod, MA, USA) according to the manufacturer's instructions. Selected proteins were also tested for masked endotoxin using a NFκB reporter assay based on HEK293 cells overexpressing TLR4, MD-2, and CD14, as previously described (29, 30).

### HPLC-MS Analysis

Purified proteins were analyzed by high-performance liquid chromatography-mass spectrometry (HPLC-MS) using a Q Exactive™ Hybrid Quadrupole-Orbitrap™ mass spectrometer (Thermo Fisher Scientific) online hyphenated to an UltiMate™ RSLC nano system (Thermo Fisher Scientific) by means of a nano-electrospray ionization source. Prior to analysis, samples were purified using Pierce™ C18 tips (Thermo Fisher Scientific) as described in the manufacturer's manual.

Two hundred and fifty nanogram of the respective protein were injected onto a Waters X Bridge Protein BEH C4 2.1 × 150 mm column with 3.5 μm particles and a pore size of 300 Å (Waters, Milford, MA, USA). Water (A) and acetonitrile (B; Sigma Aldrich) each containing 0.10% (v/v) formic acid (FA) were used as eluents. At a flow rate of 200 μL/min proteins were eluted by applying a linear gradient of 20.0–60.0% B over 3 min. The column temperature was set to 60°C. The mass spectrometer was operated in positive ionization mode. Full scans were performed at a scan range of m/z 500–1,500 at a resolution of 140,000 (at m/z = 200). The AGC-target was set to 1 × 10^6^ with a maximum injection time (IT) of 100 ms.

For data analysis, Xcalibur 3.0 (Thermo Fisher Scientific), Proteome Discoverer 1.4 (Thermo Fisher Scientific), and GPMAW (Lighthouse data, Denmark) were used.

### Molecular Dynamics and Protein Flexibility

We performed molecular dynamics simulations of wild-type Phl p 6 and the four *in silico* selected mutants to profile the respective conformational ensembles and intrinsic flexibilities. In order to achieve broad conformational sampling, we used accelerated molecular dynamics simulations (aMD). For the wild-type, the starting structure for the simulation was obtained from the available crystal structure 1NLX. We note that in this structure, only 104 of the total 111 residues of the allergen are resolved. In detail, four C-terminal residues as well as three N-terminal residues (including the starting methionine) are not resolved. For the mutants, starting structures were modeled based on the wild-type structure. The respective point mutations were introduced with the program MOE (molecular operating environment, Chemical Computing Group, Montreal, Canada), followed by local minimization. Structures were protonated at pH 7.0 with the tool protonate3d as implemented in MOE. Topology and starting coordinate files were generated with the LEaP module of AmberTools 17 (31) using the ff99SB-ILDN force field (32). Each protein was placed in a truncated octahedral TIP3P water box (33) with a minimum wall distance of 10 Å. After an elaborate equilibration protocol, consisting of subsequent relaxing, heating and cooling steps (34) short conventional molecular dynamics simulations of 100 ns length were performed to obtain acceleration parameters (35). Subsequent accelerated MD simulations were performed for 1,000 ns per system. Simulations were carried out in the NpT ensemble, using a Langevin thermostat t (36) with a collision frequency of 2 ps^−1^ to keep a constant temperature of 310 K, as well as a Berendsen barostat (37) with a relaxation time of 2 ps to keep the system at atmospheric pressure. Bonds involving hydrogen bonds were constrained with the SHAKE algorithm to allow the use of 2 fs time (38). A van der Waals cutoff of 10 Å was used and long-range electrostatics were treated with the particle-mesh Ewald method (PME) (39). All simulations were carried out with the GPU acceleration of the pmemd module of AMBER 17 on our in-house cluster. Snapshots were saved each 10 ps.

All trajectories were reweighted with a McLaurin series to the 10th order prior to analysis (35, 40). Analyses were carried out with the program cpptraj (41) of AmberTools 17 and in-house python scripts. Local flexibilities were characterized by calculating the dihedral entropies resulting from backbone torsion probability profiles (42). Structural visualizations were achieved by calculating the residues-wise differences in dihedral entropy for each system, using the wild-type as a reference and mapping the respective differences onto the respective crystal structures using the program PyMol (43).

### Protein Folding and Stability

Protein folding in solution was monitored by circular dichroism spectroscopy using a JASCO J-815 spectropolarimeter equipped with a PTC-423S Peltier-type single position cell holder (Jasco). Proteins were dissolved to 10 μM in 2 mM sodium phosphate buffer pH 7.4 and spectra were recorded from 190 to 260 nm. Thermal denaturation was monitored at 222 nm from 20 to 90°C with a temperature slope of 1°C/min.

All NMR measurements were carried out with 0.75 mM Phl p 6 in 10 mM sodium phosphate buffer pH 7.0 with 10% D_2_O. For protein assignment, standard triple-resonance methodology was employed. ^15^N HSQC spectra (44) of wild-type Phl p 6 and L89G and E39L mutants were measured in a temperature range between 20 and 70°C in steps of 5°C. Chemical shift changes due to the change in temperature were recorded using 500 μM sodium trimethylsilylpropanesulfonate (TMS) as reference substance (0 ppm at all temperatures).

### Protein Crystallization, Data Collection, and Structure Determination

For initial crystallization screening, the sitting-drop vapor-diffusion method was applied, utilizing a Hydra II Plus One (Matrix) liquid-handling system. 0.2 μL Phl p 6 mutant proteins at a concentration of 10 mg/mL were mixed with 0.2 μL screen solution from different commercial screens and equilibrated against 60 μL reservoir solution in a 96-well-plate (Art Robbins Instruments) at 293 K. Crystals were observed in condition consisting of 0.05 M zinc acetate dehydrate, 20% (w/v) polyethylene glycol (PEG) 3350 for Phl p 6 S46Y. Fine screening was carried out using different protein to drop ratio 1:1, 1:1.5, 1:2, PEG3350 concentration 10–25% in hanging drop at 293 K. Phl p 6 S46Y mutant crystals were observed in condition consisting of 0.05 M zinc acetate dehydrate, 10% (w/v) PEG3350 with a protein to drop ratio of 1:1. Micro-seeding using Phl p 6 S46Y crystals was performed to ease the crystallization of other mutants (45). The crystals were cryo-protected with 10% glycerol and flash frozen in liquid nitrogen. Diffraction datasets were collected at the ESRF beamline ID29 and processed using iMosflm (46) and Scala in the CCP4 software suite (47). The structure was solved by molecular replacement using the existing Phl p 6 structure (PDB: 1NLX). Molecular replacement was done using CCP4 software suite. Structure models were built using COOT (48) and refined using Phenix (49). Anomalous data sets were collected at the Zinc K-absorption edge and data processing was performed as described above. The two data sets were then combined and scaled using Scaleit in the CCP4 software suite. The anomalous difference was calculated using Sftools in the CCP4 software suite. Finally, the anomalous difference Fourier maps were calculated with FFT in the CCP4 software suite. The coordinates of Phl p 6 S46Y have been deposited in the Protein Data Bank under the entry code 6TRK, see Supplementary Table 1 for crystallographic data and refinement statistics.

### Endolysosomal Degradation Assay

Endolysosomal degradation assays were performed as previously described (50). Briefly, 5 μg of protein substrates (Phl p 6 WT, and its variants N16M, E39L, S46Y, and L89G) were mixed with 7.5 μg of isolated microsomal fraction from the JAWS II cell line in 50 mmol/L citrate buffer (pH 5.2, or pH 4.5) and 2 mmol/L dithiothreitol. Samples were incubated at 37°C for the indicated time points followed by 5 min denaturation at 95°C to stop the reaction. Samples were analyzed on 20% acrylamide gels by SDS-PAGE and coomassie staining followed by densitometric analysis of the full length protein using ImageJ. For Phl p 6 WT, S46Y, and L89G, the pool of generated peptides was additionally analyzed by HPLC-MS as described above for intact proteins. To identify individual peptides resulting from endolysosomal degradation, 600 ng were injected onto a self-packed 200 × 0.1 mm Hypersil GOLD™ aQ C18 (Thermo Fisher Scientific) capillary column with 3.0 μm particles. Peptides were separated at a flow rate of 350 nL/min using a linear gradient of 5.0–40.0% B in 60 min at 50°C. For MS detection, survey scans were performed for a scan range of m/z 370–2,000 at a resolution of 70,000 (at m/z = 200). The AGC target was set to 1 × 10^6^ with a maximum IT of 120 ms. The 15 most intense ions were selected for fragmentation using a normalized collision energy of 29. Data dependent MS2 scans were recorded at a resolution of 35,000 with an AGC target of 2 × 10^5^ and a maximum IT of 100 ms.

### Animal Experiments

Female 6- to 10-week-old BALB/c mice were obtained from Janvier (Le Genest-Saint-Isle, France) and maintained at the animal facility of the University of Salzburg in a specific pathogen-free environment according to local guidelines. Animal experiments were approved by the Austrian Ministry of Education, Science and Research (permit no. BMWF-66.012/0013-WF/V/3b/2017).

For initial epitope mapping and generation of T cell hybridomas, mice were immunized with 5 μg Phl p 6 adsorbed to alum (50% v/v Alu-Gel-S, Serva) by s.c. injection of 200 μL divided between two sites on the back. Immunizations were performed on days 0, 19, and 48, and mice were sacrificed 1 week later.

To assess *in vivo* immunogenicity of the different proteins and for repeated epitope mapping, mice were immunized with 10 μg of Phl p 6 or one of the mutant proteins in sterile PBS without adjuvant by means of intradermal injections at two sites on the shaved back (2 × 20 μL). Mice were immunized on days 0, 14, and 28. On day 44, mice received terminal anesthesia (100 mg/kg Ketamine + 3 mg/kg Xylazine + 3 mg/kg Acepromazine) by i.p. injection and their blood was collected from retroorbital sinus.

### Lymphocyte Cultures and Cytokines

Following terminal blood collection, mice were sacrificed by cervical dislocation. Spleens were aseptically removed and transferred into 40 mm Petri dishes containing 500 μL DPBS. Spleens were homogenized using the back of a sterile plunger from a 2 mL syringe and the suspensions were transferred into 1.5 mL tubes. After 3–5 min incubation at room temperature (until debris had settled), the monodisperse cell suspension was transferred into a 15 mL tube, pre-filled with 7 mL of ACK red blood cell lysis buffer (0.15 M NH_4_Cl, 10 mM KHCO_3_, 0.1 mM NA_2_EDTA, pH 7.2–7.4) and incubated for 7 min at RT. Tubes were filled up with 6 mL of DPBS, centrifuged for 5 min at 260 g at RT, and the pellets were washed with 5 mL of DPBS. After centrifugation, the pellets were resuspended in 10 mL warm DPBS containing 1 μM eFluor 450 cell proliferation dye (eBioscience/Thermo Fisher Scientific). After incubation for 10 min at 37°C, labeling was stopped by addition of 10% FCS. After two washing steps with DPBS, lymphocytes were re-suspended in T-cell medium (RPMI, 10% FCS, 25 mM HEPES, 2 mM L-Glu, 100 μg/mL streptomycin, 100 U/mL penicillin) and counted using a CEDEX XS Cell Analyzer (Roche). 3 × 10^5^ cells per well were incubated in U-bottom tissue culture plates with 20 μg/mL Phl p 6 or the mutant proteins, or 10 μg/mL of individual peptides from a 15 mer library (GenScript, NJ, USA) with an offset of 3 amino acids in a total volume of 150 μL per well. After 4 days, culture supernatants were removed and cells were harvested for flow cytometric analysis. Cytokine levels in supernatants were analyzed using the LEGENDplex™ Mouse TH Cytokine Panel (13-plex, BioLegend) according to the manufacturer's instructions.

### Epitope Mapping

Restimulated splenocytes were transferred to V-bottom plates and stained in DPBS for 10 min at RT with fixable viability dye eFluor 780 (1:5,000, eBioscience) and anti-mouse CD4 PerCp/Cy5.5 (1:200, BioLegend, SanDiego, USA, clone GK1.5) and finally analyzed on a Cytoflex S flow cytometer (Beckmann Coulter). Proliferating cells among live CD4+ T cells were identified by their reduced fluorescence on the eFluor 450 channel. Peptides were considered immunoreactive if the stimulation index (% proliferating cells stimulated with peptide/% proliferating cells without stimulation) was >2.

### Antibody Measurements

To determine the production of Phl p 6-specific serum IgG1 and IgG2a, a luminometric ELISA was performed at serum dilutions lying within the linear range of the assay. 96-well plates (flat white chimney; Greiner) were coated with 1 μg/mL recombinant Phl p 6 or the mutant proteins diluted in PBS (50 μL per well) overnight at 4°C. The coating solution was discarded and 200 μL of blocking buffer (2% skim milk, blotting grade, and 0.1% Tween^®^-20 in PBS) were added to each well and incubated for 1 h at RT. The plate was washed with PBS-T (PBS, 0.1% Tween-20) using an automated plate washer (Tecan 96PW Microplate Washer). Meanwhile, serum dilutions (1:100 for IgG2a and 1:100,000 for IgG1) were prepared in blocking buffer. Fifty microliter of each serum dilution was added to the wells and incubated for 2 h at 37°C in a humid chamber. After another washing step, 50 μL of the secondary antibody (goat anti-mouse IgG1-HRP diluted 1:1,000, Bio-Rad; rabbit anti-mouse IgG2a-HRP diluted 1:2,000, antibodies-online.com) were added in blocking buffer. The plate was incubated for 1 h at RT. After a final washing step, 50 μL of an ELISA BM chemiluminescence substrate (Roche) was added and after 2–3 min the luminescence was measured in relative light units (RLU) using a plate reader (Tecan inifinite 200Pro; integration time of 1,000 ms and attenuation set to automatic).

For assessment of biologically active, specific IgE in sera, a β-hexosaminidase release assay was performed. Briefly, rat basophil leukemia (RBL-2H3) cells were seeded in 96-well tissue culture plates and incubated overnight at 37°C, 5% CO_2_, 95% humidity. The next day, sera were added for passive sensitization at indicated dilutions. Background wells and wells for maximum release remained untreated. After 2 h of incubation, cells were washed three times with Tyrode's buffer containing 0.1 % BSA and incubated for 1 h with 10 ng/mL of Phl p 6 or the mutant proteins in Tyrode's buffer for stimulation. Ten microliter of a 10% Triton X-100 was added to some wells for maximum release. Supernatants containing released mediators including β-hexosaminidase were transferred to fresh plates and assay solution (4-MUG diluted in 0.1 M citrate buffer, pH 4.5) was added for 1 h. The reaction was stopped by addition of 100 μL glycine buffer (0.2 M glycine, 0.2 M NaCl, pH 10.7). Fluorescence intensity was measured on a TECAN plate reader at λ_ex_:360 nm and λ_em_:465 nm. Percentage of specific lysis was calculated by the formula (rfu – rfu_back_)/(rfu_max_ – rfu_back_)^*^100.

Cell-bound IgE was measured *ex vivo* using a basophil activation test (BAT). On the day of sacrifice, blood was taken from the retro orbital sinus and mixed with 1/10 vol. of Li-Heparin (1.5 mg/mL in DPBS) to prevent coagulation. Thirty microliter of these blood samples were mixed with 30 μL of RPMI with an equimolar mix of Phl p 6 WT, L89G, and S46Y and incubated for 2 h at 37°C, 5% CO_2_, 95% humidity. Final concentration of Phl p 6 in stimulated wells was 10 ng/mL. After 2 h, stimulation was stopped by putting the plates on ice, and all subsequent steps were performed with ice-cold solutions and samples were kept on ice. Samples were washed with 120 μL FACS buffer (PBS, 1% BSA, 2 mM EDTA) and cell pellets (5 min centrifugation at 260 g) were resuspended in 30 μL of FACS buffer containing anti-IgE-FITC (BioLegend, clone RME-1, 1:200), anti-CD4-PerCp-Cy5.5 (BioLegend, clone GK1.5, 1:200), anti-CD19-PE/Cy7 (BioLegend, clone 6D5, 1:200), and anti-CD200R-APC (eBioscience/Thermo Fisher Scientific, clone OX110, 1:200). Samples were stained for 20 min on ice and washed with 100 μL FACS buffer. After 5 min centrifugation at 260 g, pellets were resuspended in 150 μL red blood cell lysis buffer (eBioscience/Thermo Fisher Scientific) and incubated for 5 min at RT. Cells were centrifuged for 5 min at 400 g and pellets were washed two times with FACS buffer. Finally, cell pellets were resuspended in 50 μL FACS buffer and analyzed on a FACS Canto II flow cytometer (BD Biosciences). Basophils were gated as IgE^high^ CD19^neg^ CD4^neg^ cells and activation status was assessed by the median fluorescence intensity of CD200R as previously described (51).

### Generation of BMDCs and Phl p 6–Specific T Cell Hybridomas

For generation of bone marrow-derived dendritic cells (BMDCs), bone marrow was harvested from femur and tibia of BALB/c mice. Ten milliliter of a 2 × 10^5^ cells/mL bone marrow cell suspension were plated into non-cell culture treated petri dishes and incubated in the presence of murine GM-CSF (Immunotools, Germany) for 6–8 days. On day 3, 10 mL of GM-CSF containing culture medium (RPMI1640 supplemented with 20 ng/mL GM-CSF, 100 U/mL penicillin, 100 μg/mL streptomycin, 2 mM L-Glutamine, 10% FCS and 50 μM 2-mercaptoethanol) was added to each well, and on day 5, 10 mL medium was replaced.

To generate Phl p 6-specific T cell hybridomas, splenocytes from Phl p 6 immunized mice were cultured in Opti-MEM + GlutaMAX (Gibco), 5% FCS, 100 U/mL penicillin, 100 μg/mL streptomycin, with the immunodominant peptide 31 (AA 92–106) at a final concentration of 3 μg/mL. One week later, dead cells and peptides were removed by ficoll density gradient centrifugation and cells were incubated with 20 U/mL recombinant human IL-2 for another week. After removal of IL-2 by washing, T cells were co-cultured with syngeneic irradiated feeder cells (splenocytes from naïve BALB/c mice) with 6 μg/mL of the peptide for 8 days. On day 8, T cells were harvested and fused with mouse thymus lymphoma cell line BW5147.G.1.4 (ATCC) according to standard protocols for generation of T cell hybridomas (52).

### Processing of Phl p 6 and Phl p 6 Mutants by BMDCs and Stimulation of Phl p 6–Specific T Cells

Bone marrow-derived dendritic cells, generated by culturing bone marrow cells in the presence of GM-CSF, were incubated in 96-well U-bottom plates for 16–64 h in the presence of 20 μg/mL Phl p 6 WT, S46Y, or L89G in GM-CSF containing culture medium (RPMI1640 supplemented with 20 ng/mL GM-CSF, 100 U/mL penicillin, 100 μg/mL streptomycin, 2 mM L-Glutamine, 10% FCS and 50 μM 2-mercaptoethanol) in triplicates. Control wells received medium alone. After washing the cells 2 times with DPBS, 4 × 10^5^ Phl p 6-specific T cell hybridoma cells were added in DMEM, 4 mM L-Glu, 10% FCS and co-cultured with protein-loaded BMDCs for another 24 h. Standard wells contained BMDCs that were pulsed with different concentrations of peptide 92–106 during the 24 h of co-culture. Culture supernatants were removed and analyzed for IL-2 production as a correlate for T cell activation using an ELISA MAX mouse IL-2 set (BioLegend). IL-2 values measured in supernatants from the wells containing BMDCs pulsed with proteins for different time periods were transformed into μM peptide equivalents by interpolating in a standard curve generated from the values obtained from hybridomas co-cultured with BMDCs at indicated peptide concentrations.

### Statistics

Statistical differences between groups were analyzed by one-way ANOVA followed by Tukey's *post-hoc* test unless otherwise indicated (Prism 7, GraphPad Software). Data are expressed as means ± SEM (*P*-value range is indicated: ^*^P < 0.05, ^**^P < 0.01, ^***^P < 0.001, and ^****^ *P* < 0.0001).

## Results

### Identification of Dominant T Cell Epitopes of Phl p 6

**Figure 1:**
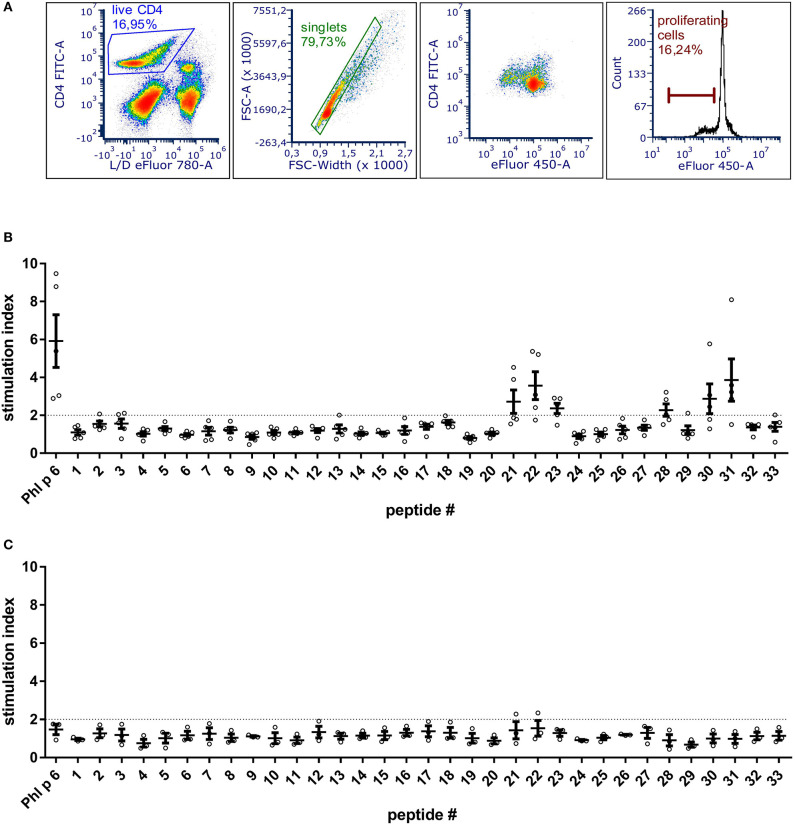
BALB/c mice immunized with Phl p 6 display two immunodominant T cell epitope regions. Splenocytes from Phl p 6-immunized or naïve control mice were stained with eFluor 450 proliferation dye and restimulated for 3 days *in vitro* with Phl p 6 or 33 individual peptides covering its entire sequence (15mer with 3AA overlap). Control samples were stimulated with medium alone. Proliferation of splenocytes was determined by flow cytometry. Live CD4 cells were gated followed by exclusion of doublets. Proliferating T cells were identified by their reduced levels of eFluor 450 dye **(A)**. Data from Phl p 6-immunized **(B)** or naïve control mice **(C)** are shown as stimulation index (% proliferation peptide/% proliferation medium). A stimulation index above 2 (dotted line) was considered as significant. Data are shown for individual mice (*n* = 5) and as means ± SEM.

To be able to exclude relevant T cell epitopes from the mutation process, the dominant T cell epitopes of Phl p 6 were identified by culturing splenocytes from Phl p 6-immunized mice together with 33 overlapping peptides covering its entire sequence (15mers, 3AA overlap). By determining the proliferation rate of CD4+ T cells, two major (peptides 22 and 31) and one minor (peptide 28) T cell epitopes were found (Figure 1). Sequences of the respective T cell epitopes are shown in Table 1.

**Table 1:** Phl p 6 T cell epitopes mapped in BALB/c mice.

**Peptide #**	**Sequence**	**AA position**
22	DEVYNAAYNAADHAA	65–79
28	KYEAFVLHFSEALRI	82–96
31	SEALRIIAGTPEVHA	92–106

### *In silico* Mutagenesis of Phl p 6

In an unbiased approach, stabilizing and destabilizing single point mutations were calculated using the MAESTRO stability prediction software (25). The dominant T cell epitopes (65–79 and 92–106, Figure 1 and Table 1) were excluded from the mutation process and the candidates were ranked by predicted changes in free energy (ΔΔG values, Tables 2, 3). We found surface exposed as well as buried amino acid exchanges among the top 10 stabilizing mutations, and chose two of each for further analysis (N16M, D52L, E39L, S46Y). On the contrary, the top 10 predicted mutations with destabilizing effect all targeted buried residues, primarily exchanging hydrophobic amino acids within the protein core, potentially leading to complete unfolding as indicated by the high ΔΔG values. Furthermore, the number one candidate mutation was located close to an immunodominant T cell epitope, potentially altering a proteolytic cleavage site and thus abolishing its processing and presentation. Therefore, we selected the mutant ranked second (L64G) and performed another scan restricted to surface exposed residues, resulting in more moderate ΔΔG values. Here, the top two proposed mutations were exchanges to prolines within the alpha helical structure of Phl p 6. As prolines are helix breakers, these mutations would dramatically alter the overall 3D structure of the mutant. Thus, we chose mutant L89G (ranked third) for expression and characterization.

**Table 2:** In silico prediction of Phl p 6 stabilizing mutations using MAESTRO algorithm.

**Mutant**	**ΔΔG_pred**	**c_pred**	**ASA**
N16M	−1.4691	0.886	Buried
D52L	−1.3471	0.893	Surface
N16F	−1.3469	0.970	Buried
N16Y	−1.3052	0.982	Buried
E39L	−1.2817	0.890	Surface
S46Y	−1.2734	0.955	Buried
N16L	−1.2711	0.816	Buried
S46F	−1.2654	0.930	Buried
M1R	−1.2512	0.871	Surface
N16W	−1.2431	0.964	Buried

*Stabilizing mutations selected for expression and further analysis are marked in red. ΔΔG_pred, predicted change in free energy [kcal/mol]. Negative values indicate a stabilizing mutation; c_pred, confidence estimation (0–1) of the prediction; 1, high confidence; ASA, accessible surface area: <30% = buried, >30% = surface exposed*.

**Table 3:** In silico prediction of Phl p 6 destabilizing mutations using MAESTRO algorithm.

**Surface exposed**			**Buried**		
**Mutant**	**Δ*Δ*G_pred**	**c_pred**	**Mutant**	**Δ*Δ*G_pred**	**c_pred**
L89P	2.0159	0.875	F91G	5.1619	0.726
A17P	1.9855	0.831	L64G	5.0143	0.757
L89G	1.9812	0.864	F19G	4.7531	0.727
A41D	1.9513	0.864	F87G	4.6827	0.735
D82S	1.8896	0.889	L60G	4.6052	0.758
A41P	1.7979	0.831	F91D	4.5717	0.748
D82P	1.7622	0.884	L64E	4.5235	0.807
A41G	1.7168	0.885	L11D	4.4678	0.772
A41N	1.6797	0.855	F38G	4.4658	0.743
A24P	1.6424	0.820	F42G	4.4645	0.733

*Destabilizing mutations selected for expression and further analysis are marked in green. ΔΔG_pred, predicted change in free energy [kcal/mol]. Positive values indicate a destabilizing mutation. c_pred, confidence estimation (0–1) of the prediction. 1, high confidence*.

### Expression and Characterization of Phl p 6 and Its Variants

**Figure 2:**
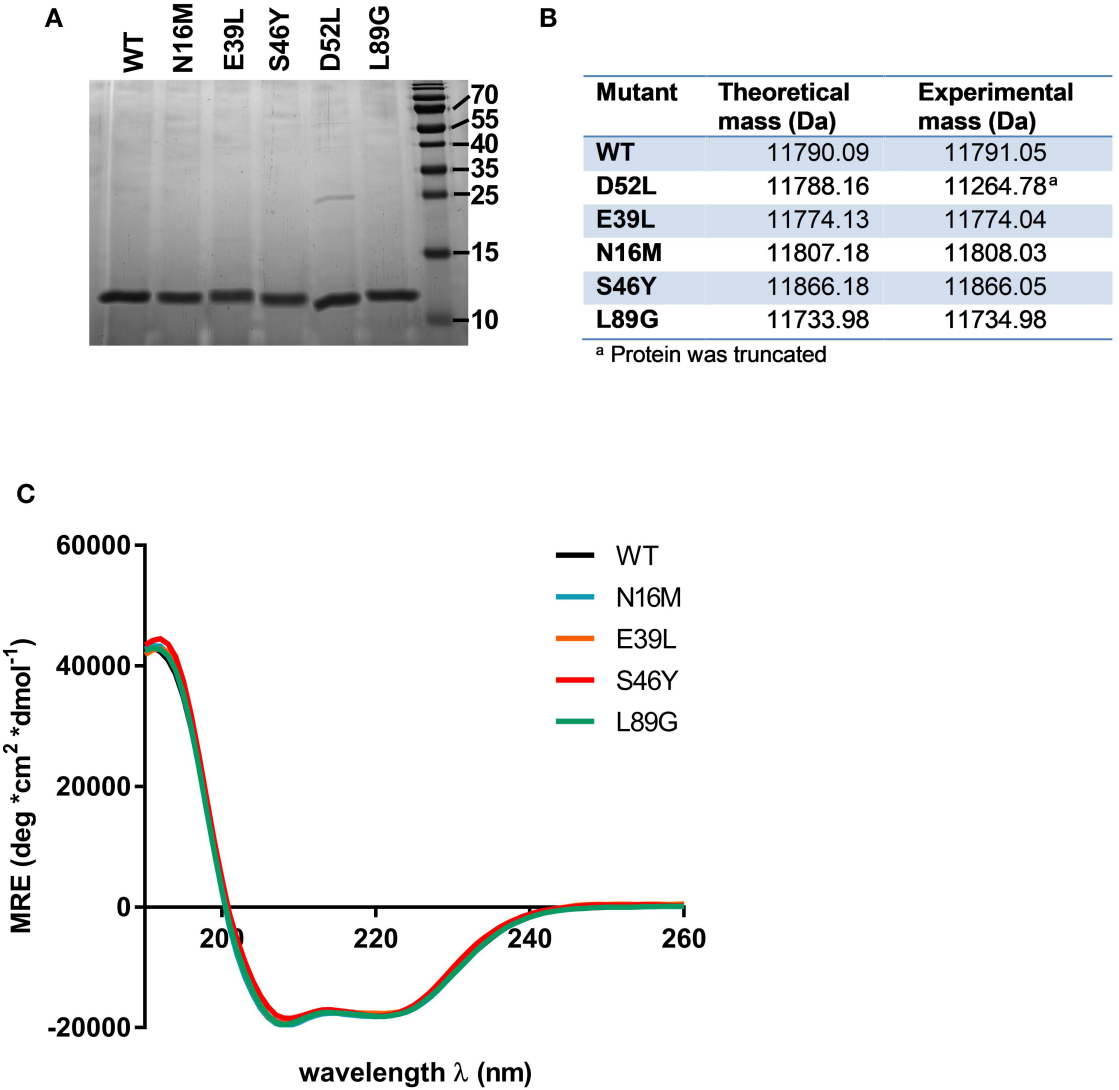
Stabilized and destabilized variants of Phl p 6 display the characteristic alpha helical fold of the wild-type protein. Recombinant Phl p 6 (WT) and its mutants (D52L, E39L, N16M, S46Y, L89G) were expressed in *E. Coli*. Purified proteins were analyzed for purity by SDS-PAGE **(A)**. Incorporation of mutations and correct size was assessed by HPLC-MS, comparing theoretical and experimental average mass **(B)**. Full CD spectra measured at room temperature, pH 7.4 confirmed very similar folding of the WT and mutant proteins **(C)**. CD data are shown as mean residual ellipticity (MRE).

Proteins were expressed tag-free in *E. coli* with the exception of mutant L64G, which we were unable to produce, most likely due to its high degree of destabilization, impeding proper folding. Subsequently, proteins were purified by hydrophobic interaction chromatography followed by anion exchange chromatography and finally by size exclusion chromatography. The final purity was determined by SDS-PAGE using a 15% acrylamide gel and Coomassie staining (Figure 2A). After endotoxin removal, all preparations contained <1 pg LPS per μg of protein according to *Limulus* amebocyte lysate assay. Proteins used for immunization of mice were additionally tested for masked LPS in a cell-based NF-κB-luciferase reporter gene assay using HEK293 cells overexpressing the LPS receptor subunits TLR4, CD14, and MD-2. LPS content in Phl p 6 WT and the mutant S46Y were below the detection limit of 0.0025 pg/μg protein; the L89G preparation contained <0.1 pg/μg. To confirm the correct amino acid substitutions in the mutants, the exact mass of the proteins was determined by means of mass spectrometry. The obtained spectra were de-convoluted using the Xtract function of Xcalibur 3.0 software (Thermo Fisher Scientific) and the experimentally determined mass was compared to the theoretical mass of the WT and the mutants (Figure 2B).

CD spectra recorded from 190 to 260 nm confirmed that the overall three dimensional structure of the proteins was not altered by introduction of the stabilizing or destabilizing mutations (Figure 2C).

Mutant D52L turned out to be truncated and was excluded from further analysis.

**Figure 3:**
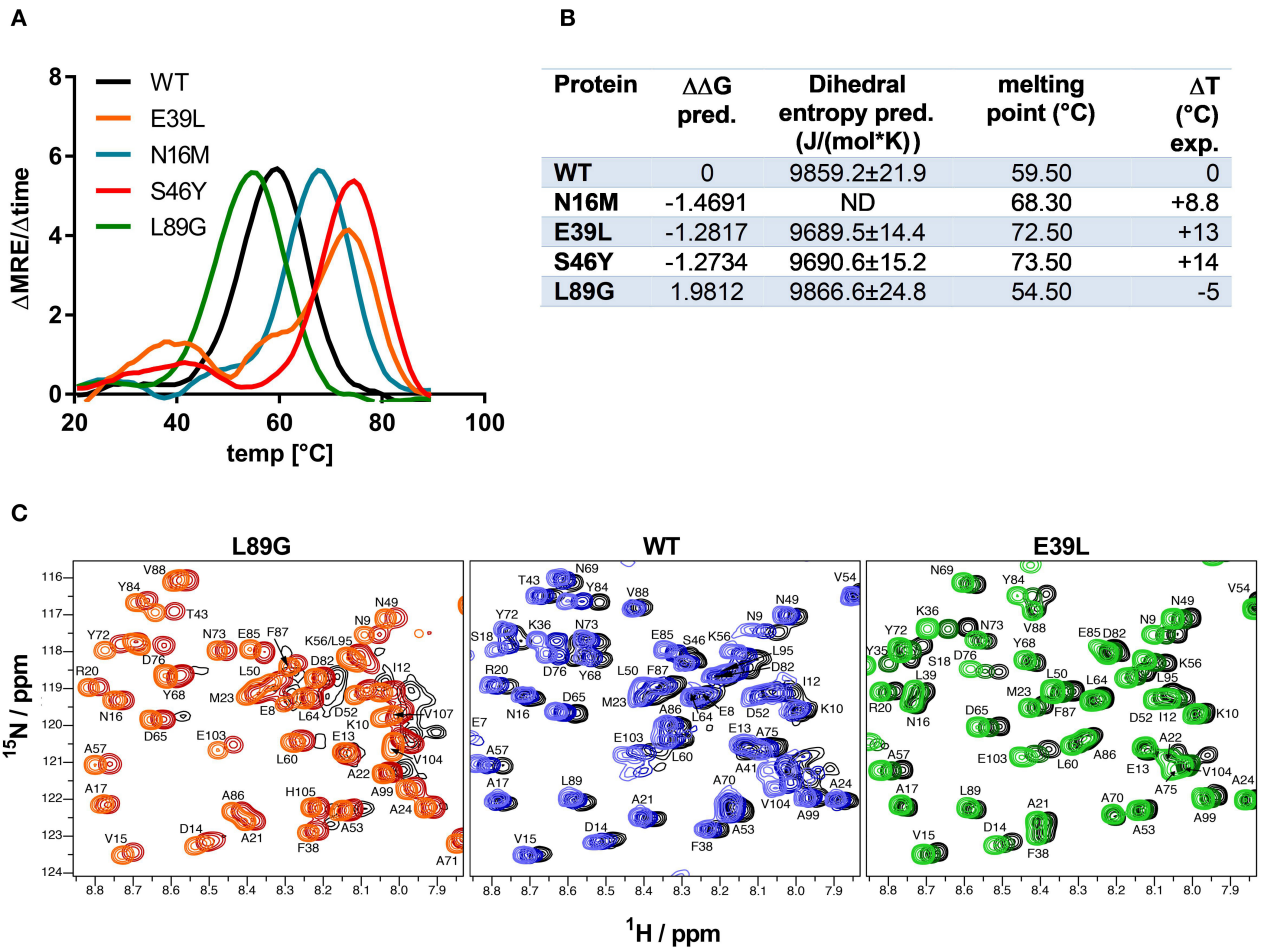
Predicted changes in free enthalpy by introducing stabilizing or destabilizing single point mutations in Phl p 6 correlate with thermal stability. CD spectroscopy of recombinant Phl p 6 (WT) and its mutants (E39L, N16M, S46Y, L89G) was performed at 222 nm using a temperature ramp from 20 to 90°C. The melting point of each protein was calculated as the inflection point (peak of the first derivative) of the mean residual ellipticity (MRE) curve **(A)**. Predicted (pred.) changes in free enthalpy (ΔΔG) and dihedral entropy were compared to the experimentally (exp.) determined thermal melting point. The melting point shifts (ΔT) compared to the wild-type protein are shown. ND, not done **(B)**. Protein backbone flexibility at different temperatures was measured by NMR. Sections of backbone amide ^1^H^15^N HSQC NMR spectra of Phl p 6 recorded at 700 MHz are shown. From left to right: destabilized mutant L89G at 35°C (orange), 40°C (red) and 45°C (black), wild-type at 35°C (light blue), 40°C (dark blue) and 45°C (black), and stabilized mutant E39L at 35°C (light green), 40°C (dark green) and 45°C (black). Backbone amide NH cross peaks are labeled **(C)**.

**Figure 4:**
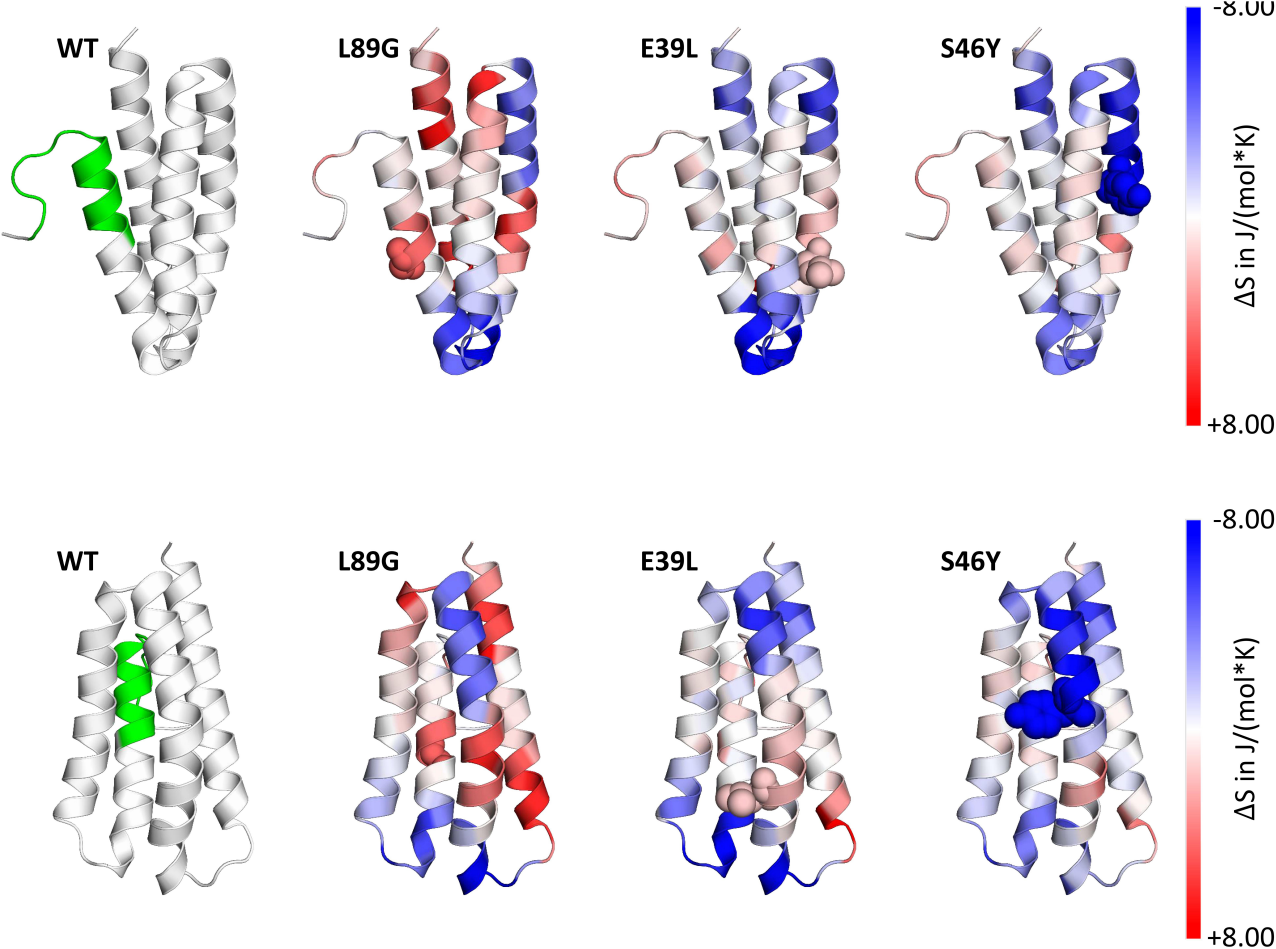
Accelerated Molecular Dynamics (aMD) simulations confirmed enhanced flexibility of destabilized mutant L89G and reduced flexibility of the stabilized mutants E39L and S46Y compared to wild-type (WT) Phl p 6. Dihedral Entropies were calculated from aMD simulations to predict localized flexibility. The immunodominant epitope 92–106 is shown in green. Red indicates increased flexibility compared to the WT molecule and blue indicates reduced flexibility. Top and bottom panels show the same molecules in different orientations. Point mutations are displayed as spheres.

Stability of the proteins was assessed by CD (Figure 3A) and NMR (Figure 3C, WT, L89G, and E39L only). As predicted by MAESTRO, mutants E39L, N16M, and S46Y displayed an increased thermal stability compared to the WT protein (ΔT +8.8 to +14°C) whereas the destabilized mutant L89G was less heat stable (ΔT −5°C). The MAESTRO predictions were also confirmed by an accelerated molecular dynamics (aMD) simulation, with the exception of N16M, which was predicted to be extremely unstable in aMD simulations (Figures 3B, 4).

The increased fold stability of E39L and the decreased fold stability of L89G were also experimentally confirmed by NMR. Upon heating, the signal intensities of L89G began to diminish at lower temperatures, whereas the signal intensities of E39L began to diminish at higher temperatures compared to WT Phl p 6 (Figure 3C).

**Figure 5:**
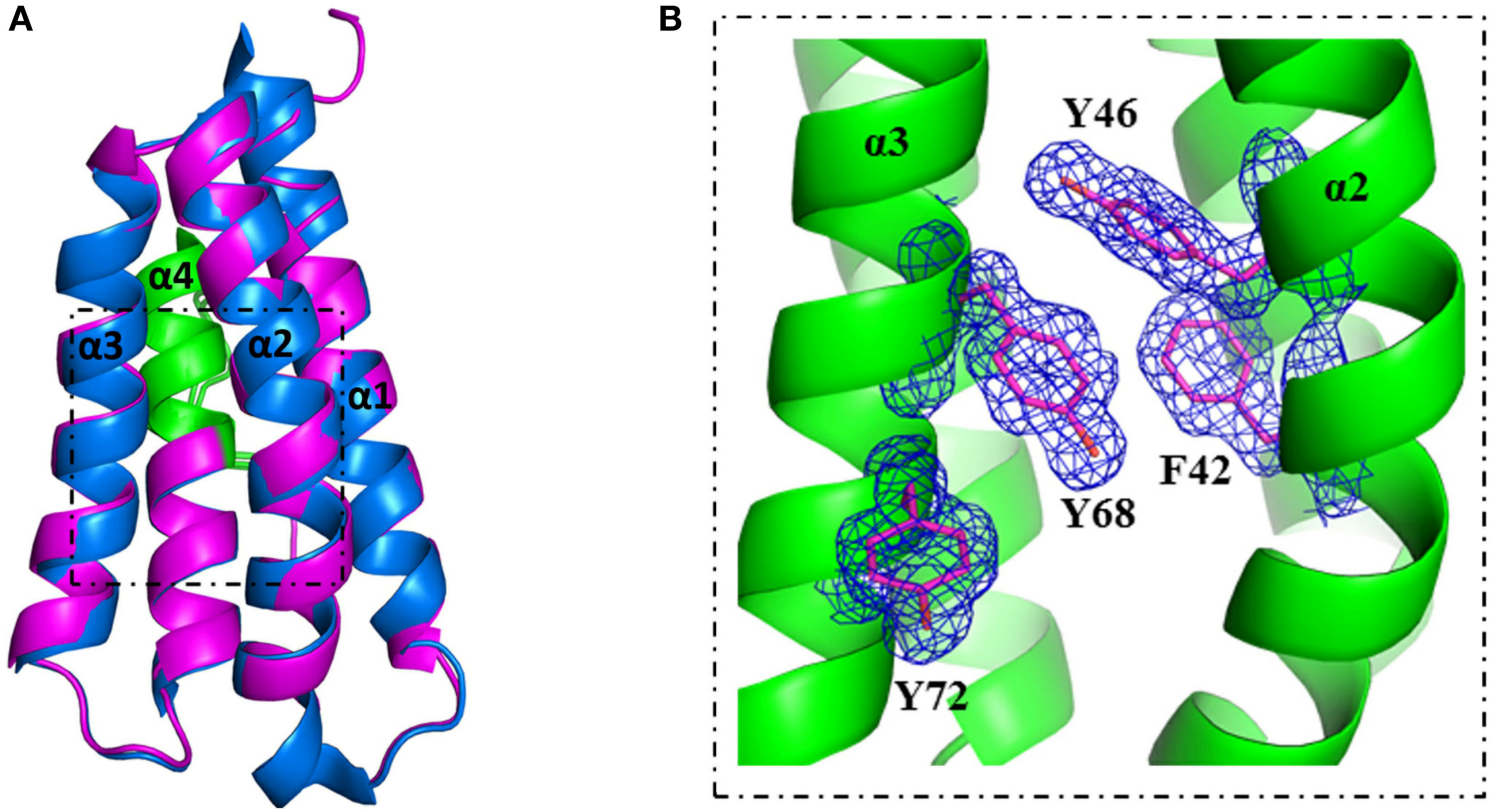
Crystal structure of stabilized mutant S46Y revealed stabilization of the hydrophobic core. The crystal structure of Phl p 6 mutant S46Y was solved at 1.6 Å resolution (PDB: 6TRK) and structurally aligned with Phl p 6 wild-type (PDB: 1NLX). Mutant S46Y (magenta) has an overall high structural similarity (Cα-RMSD: 0.55 Å) to the wild-type protein (blue). The immunodominant epitope (92–106) is labeled in green **(A)**. A close up view of the hydrophobic core shows that the Tyr46 point mutation complements the aromatic stacking and thereby improves both the stability of helix α2 (harboring Tyr46) as well as the hydrophobic core interaction of the four-helix bundle architecture α1–α4 of Phl p 6. The density is displayed as a 2Fo-Fc difference map contoured at σ = 1.5 **(B)**.

A more detailed analysis with X-ray crystallography of mutant S46Y showed that the 3-dimensional structure was remarkably similar to that of the WT protein. The superimposition of the crystal structures of S46Y with Phl p 6 WT revealed that the overall fold is identical, with a total root-mean-square deviation of 0.42 A. As shown in Figure 5, the introduction of Tyr46 in the α2 helix, pointing into the central hydrophobic cavity, contributes to hydrophobic interactions with Phe42 within the α2 helix (and also Tyr68 in the neighboring α3 helix). These intra- and inter-helical hydrophobic interactions contribute to the overall structural stabilization.

### Fold-Stability of Phl p 6 Affects Proteolytic Processing and Presentation of Antigenic Peptides to T Cells

**Figure 6:**
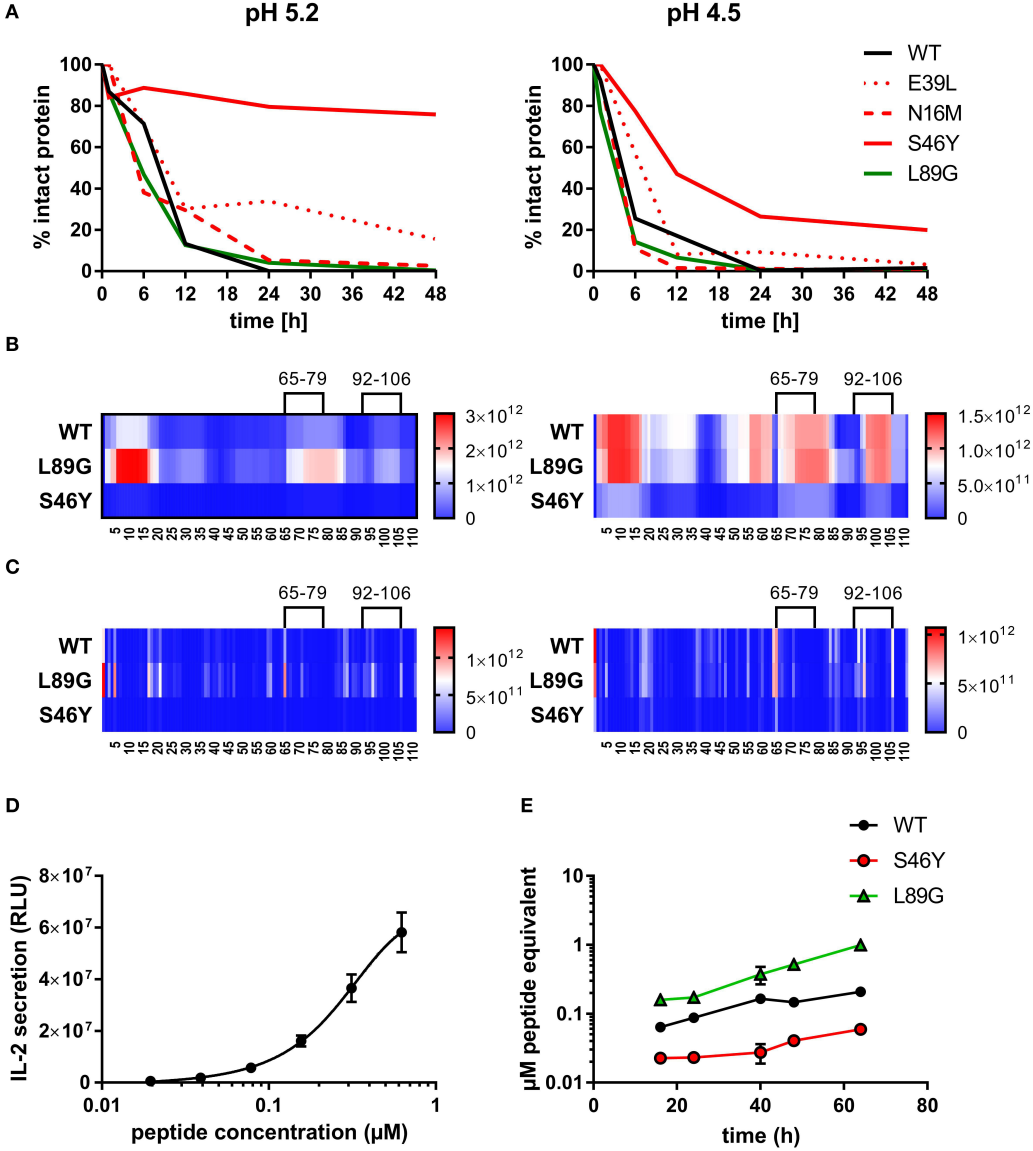
(De)stabilization affects proteolytic processing and presentation of the immunodominant epitope 92–106 of Phl p 6. Wild-type (WT) Phl p 6, and the different mutants (E39L, N16M, S46Y, L89G) were incubated with endolysosomal preparations from JAWS-II cell line for 0, 6, 12, 24, and 48 h at pH 5.2 (left) and 4.5 (right). The percentage of remaining intact protein was estimated by SDS-PAGE and Coomassie staining **(A)**. Peptides generated from Phl p 6 WT, L89G, and S46Y after 1, 3, 6, and 12 h were additionally analyzed by mass spectrometry. Peptides **(B)** and their respective cutting sites **(C)** generated earlier during the proteolytic processing are colored in red, whereas peptides that were not detected during the whole experiment are colored in blue. Presentation of the immunodominant peptide 92–106 was analyzed *in vitro* by co-culturing bone marrow derived dendritic cells (BMDC) with peptide specific T cell hybridoma cells. IL-2 secretion from hybridoma cells directly correlates with the amount of peptide added to the assay. Data are shown as means ± SD (*n* = 3) of relative light units (RLU) of a luminometric ELISA **(D)**. Using IL-2 secretion from T cell hybridomas as read-out, presentation of peptide 92–106 by BMDCs incubated with 20 μg/mL WT Phl p 6 or mutants L89G, and S46Y was assessed over time. Data are shown as μM peptide equivalents (the concentration of peptide needed to stimulate equivalent IL-2 secretion) **(E)**.

**Figure 7:**
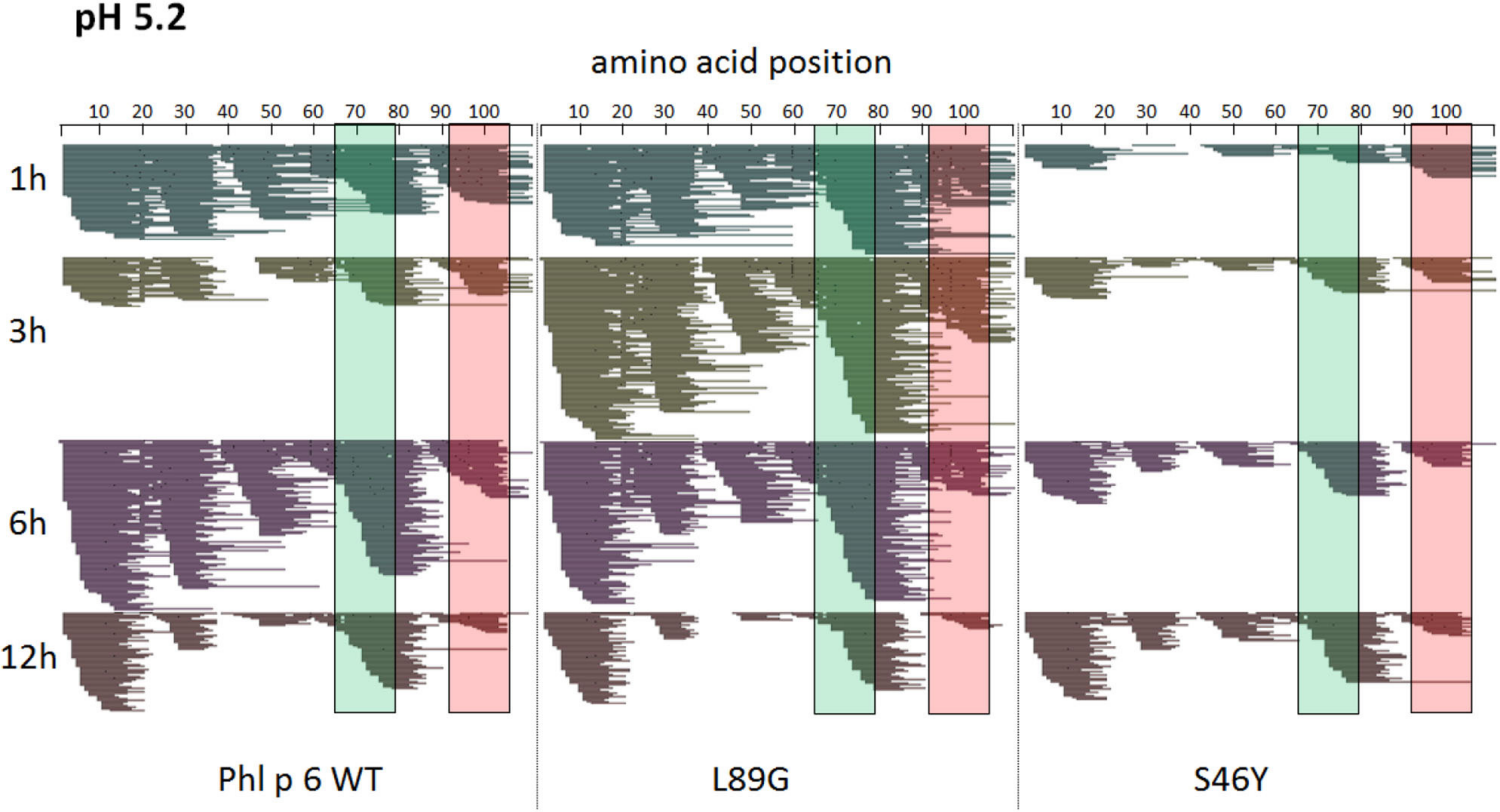
A similar pattern of peptides is generated by endolysosomal degradation of Phl p 6 wild-type (WT) and the mutants L89G and S46Y. Proteins were incubated with an endolysosomal extract from JAWS-II cell line at pH 5.2. Peptides were identified by LC-MS/MS and are displayed color-coded corresponding to different incubation times (1, 3, 6, and 12 h). Amino acid regions matching the immunodominant epitopes 65–79 and 92–106 of Phl p 6 are highlighted in green and red, respectively.

**Figure 8:**
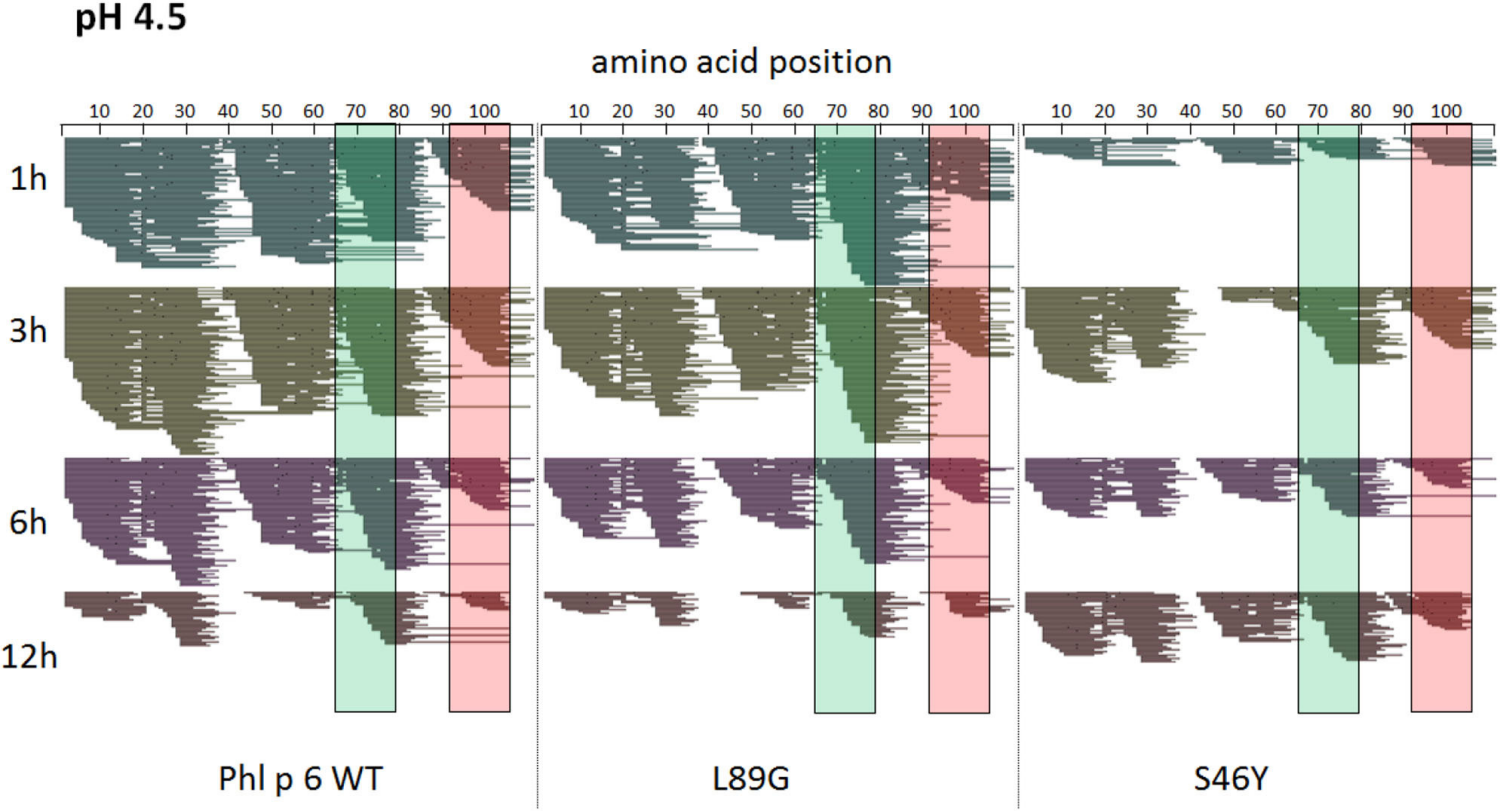
A similar pattern of peptides is generated by endolysosomal degradation of Phl p 6 wild-type (WT) and the mutants L89G and S46Y. Proteins were incubated with an endolysosomal extract from JAWS-II cell line at pH 4.5. Peptides were identified by LC-MS/MS and are displayed color-coded corresponding to different incubation times (1, 3, 6, and 12 h). Amino acid regions matching the immunodominant epitopes 65–79 and 92–106 of Phl p 6 are highlighted in green and red, respectively.

To assess how the observed changes in thermal stability affect proteasomal degradation and thus antigen presentation, the different proteins were incubated with an endolysosomal extract of the JAWS II dendritic cell line. Proteolytic degradation of the proteins was determined by SDS-PAGE and Coomassie staining. At a pH of 5.2, the destabilized mutant L89G showed the fastest degradation. Of the stabilizing mutations, N16M did not increase proteolytic stability but resulted in even faster degradation compared to the WT at some early time-points. In contrast, E39L and S46Y clearly showed delayed proteolytic processing, with S46Y being the most stable protein (Figure 6A, left panel). As expected, at a more acidic pH, proteolysis was more efficient but the picture in general stayed the same (Figure 6A, right panel). As the bulk proteolysis occurred within the first 12 h, we repeated the experiment with the WT protein, the destabilized protein L89G and the most stable protein S46Y, and followed the degradation kinetics within the first 12 h by quantitating the generated peptides by mass spectrometry. As shown in Figure 7 (pH 5.2) and Figure 8 (pH 4.5), the general pattern of generated peptides was very similar for the three proteins. However, the processing speed was quite different (Figure 6B). Confirming SDS-PAGE analysis, the N terminus of L89G was processed much faster compared to the WT protein, and also the regions containing the immunodominant epitopes 65–79 and 92–106 were degraded faster. Interestingly, we observed more efficient cutting right next to the N-terminus (position 89–91) of the immunodominant epitope in mutant L89G compared to the WT protein (Figure 6C). This fits to data from aMD analysis that predicted this region to be more flexible in L89G compared to the WT (Figure 4) providing an explanation for its higher accessibility to endosomal proteases. We then used T cell hybridomas specific for peptide 92–106 to detect pMHCII complexes on the surface of BMDCs. BMDCs were co-cultured with this T cell hybridoma together with different concentrations of the peptide 92–106. As shown in Figure 6D, the amount of IL-2 secreted by the hybridoma cells was proportional to the peptide concentration. Thus, IL-2 secretion by T cell hybridomas could be used as readout for the amount of pMHCII complexes presented on the surface of BMDCs. Using peptide pulsed BMDCs as standard curve, we calculated the pMHCII concentration (as μM peptide equivalents) from BMDCs incubated with the different proteins for 16–64 h (Figure 6E). Confirming the degradome data, peptide 92–106 was much faster and more efficiently processed from the destabilized mutant L89G compared to the WT or S46Y. After 64 h, the processing and presentation of peptide 92–106 was 3.3-fold more efficient from WT Phl p 6 and 16.7-fold more efficient from L89G compared to S46Y.

### Stabilized Mutant S46Y Shows Less IgE Binding and Induces Higher IgG2a Compared to the Wild-Type Protein and Destabilized Mutant L89G

**Figure 9:**
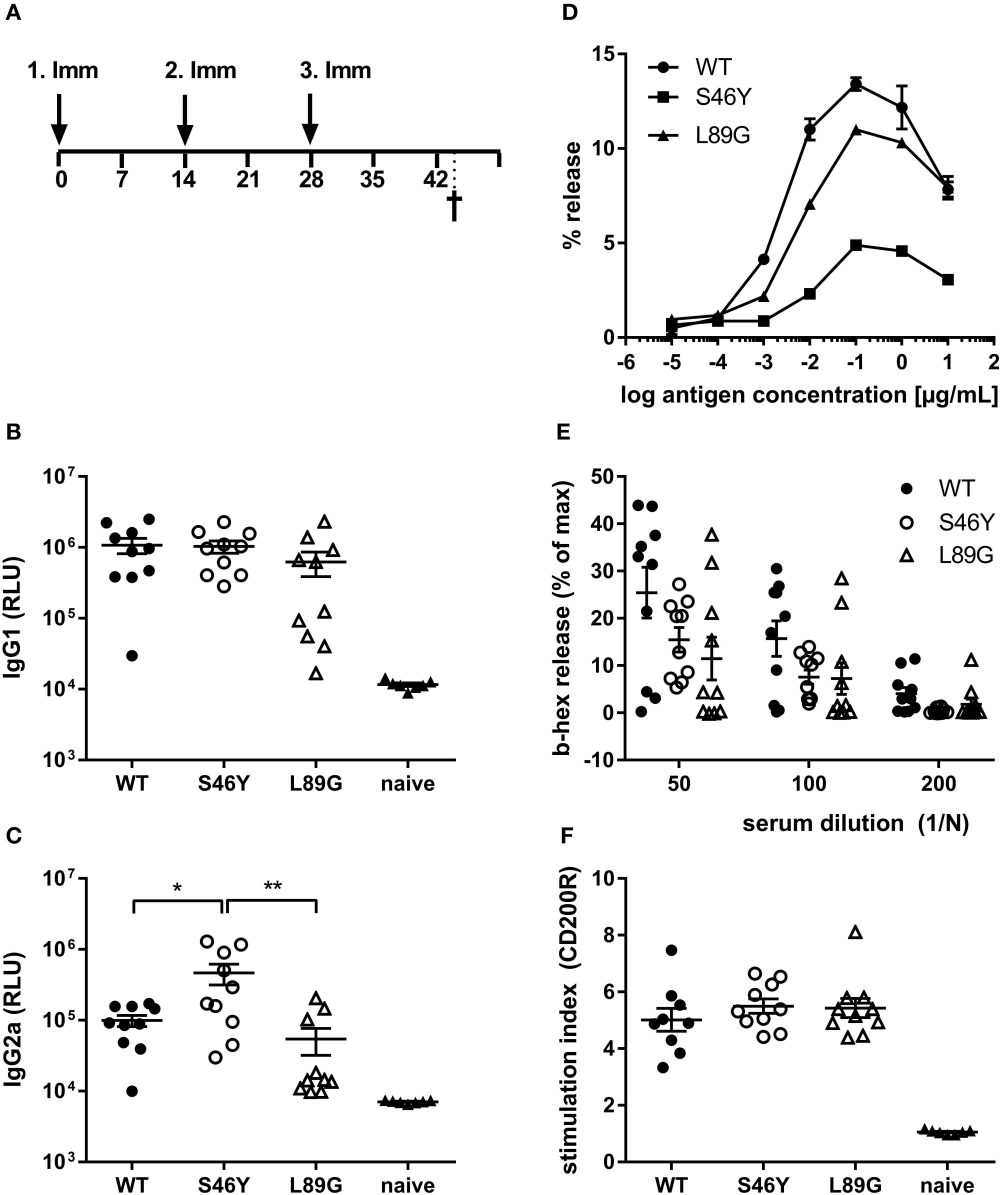
Stabilization of Phl p 6 shifts immune responses toward T helper 1 reactions. For assessment of *in vivo* immunogenicity, BALB/c mice were immunized three times at 2-week intervals by intradermal injections and sacrificed 2 weeks after the last immunization **(A)**. Phl p 6-specific serum IgG1 **(B)** and IgG2a **(C)** were measured by luminometric ELISA at serum dilutions of 1:100,000 and 1:100, respectively, lying within the linear range of the assay. Data are shown as means ± SEM (*n* = 10) of relative light units (RLU). IgE crosslinking capacity of the different proteins was tested using RBL cells loaded with high titered Phl p 6 WT specific IgE. Increasing concentrations of the different proteins were added and cross-linking induced beta-hexosaminidase (b-hex) release is presented as percentage of maximum release induced by Triton-X100 cell lysis (*n* = 3, means ± SD) **(D)**. Biologically active IgE in sera from immunized mice (WT, L89G, S46Y) were measured by incubating RBL cells with serum (*n* = 10, means ± SEM) at indicated dilutions, followed by cross-linking with WT Phl p 6 **(E)**. Cell bound IgE in blood from immunized (WT, L89G, S46Y) and naïve control mice (*n* = 10) was measured by basophil activation test after *in vitro* stimulation of whole blood samples with 10 ng of an equimolar mixture of WT, S46Y, and L89G protein. IgE cross-linking induced upregulation of activation marker CD200R was measured by flow cytometry. Data are shown as means ± SEM of stimulation indices (SI) **(F)**. **P < 0.01, *P < 0.05.

**Figure 10:**
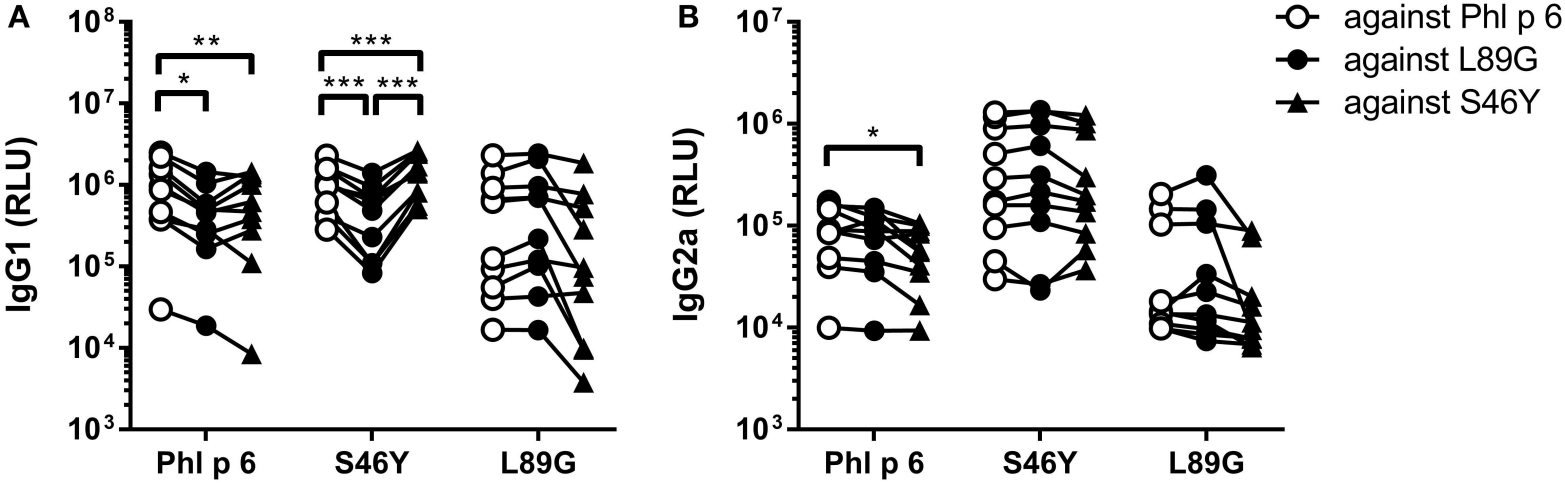
Antibodies induced by immunization with L89G and S46Y bind WT Phl p 6 with similar efficacy compared to the homologous protein. Sera (*n* = 10) were analyzed for their reactivity against Phl p 6 WT and the different mutant proteins using a luminometric ELISA. IgG1 **(A)** and IgG2a **(B)** data are shown as relative light units (RLU). Individual serum samples tested against the different proteins are connected by lines. Statistical differences were assessed by two-way RM ANOVA followed by Tukey's *post-hoc* test. ^*^P < 0.05, ^**^P < 0.01, ^***^P < 0.001.

To investigate how altered fold stability affects immunogenicity *in vivo*, we immunized BALB/c mice with Phl p 6 WT, stabilized mutant S46Y, and destabilized mutant L89G by intradermal immunization (Figure 9A). To detect a modulation of immune polarization induced solely by differences in the stability of the proteins, we did not use any adjuvant. Two weeks after the third immunization, Phl p 6-specific antibodies were measured by ELISA. Mice immunized with mutant S46Y displayed significantly higher IgG2a titers compared to the WT and L89G. In contrast, L89G induced the lowest IgG1 as well as IgG2a titers (Figures 9B,C). To rule out that this effect was due to changes in B cell epitopes induced by conformational changes in the mutated proteins, we tested the reactivity of sera from mice immunized with S46Y or L89G also against the homologous protein (used for immunization). As shown in Figure 10, antibodies raised against the mutant proteins recognized the WT molecule with similar efficacy compared to the homologous molecule. As expected, antibodies raised against the stabilized protein showed the lowest reactivity against the destabilized protein and *vice versa*. Similarly, high titered IgE sera raised against the WT protein showed intermediate reactivity with L89G and the lowest reactivity with S46Y (Figure 9D). Taken together, the differences in Phl p 6-specific IgG2a titers induced by the different mutant proteins cannot be explained by conformational changes affecting B cell epitopes, but reflect changes in the immunogenicity of the proteins.

Although the proteins differed in their IgG2a inducing potential, all groups showed similar levels of serum IgE (RBL assay, Figure 9E) or cell bound IgE (basophil activation test, Figure 9F) after *in vitro* restimulation with WT Phl p 6 or an equimolar mixture of WT, S46Y, and L89G, respectively.

### Changing the Fold Stability of Phl p 6 Does Not Alter the Repertoire of Presented Peptides *in vivo*

**Figure 11:**
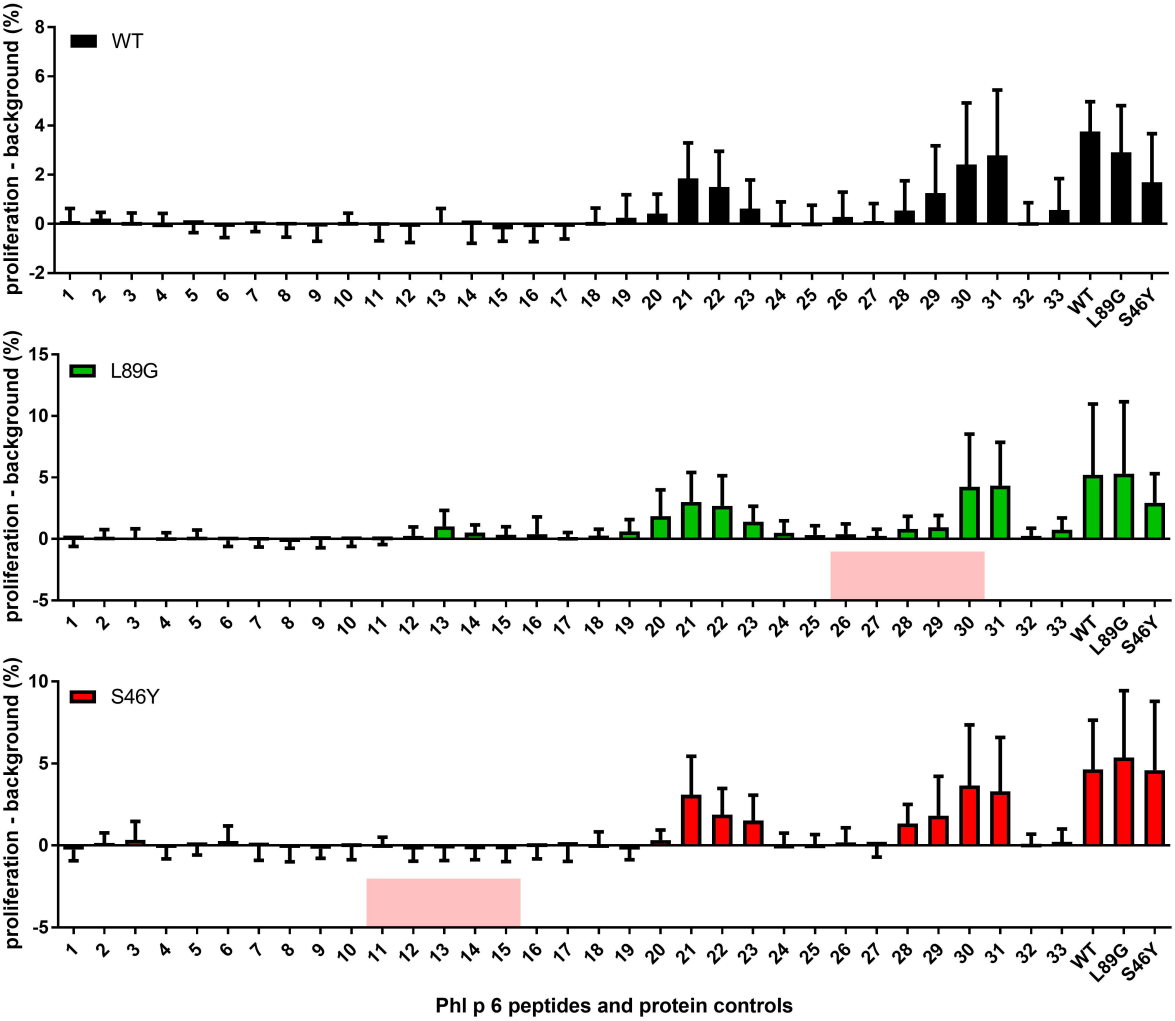
The repertoire of *in vivo* presented peptides remains largely unaffected by the fold-stability of the protein. Epitope specific CD4 T cell responses were mapped in BALB/c mice (*n* = 10) immunized with Phl p 6 wild-type (WT), or mutants L89G or S46Y. Splenocytes were restimulated with individual 15mer overlapping peptides covering the full sequence of the WT protein, or the full length proteins. Proliferation was assessed by flow cytometry of eFluor 450 labeled cells. Data are shown as means ± SEM of the percentage of proliferating CD4 T cells minus the background proliferation (medium control). Background levels for WT, L89G, and S46Y were 0.59% ± 0.63, 1.40% ± 2.10, and 1.20% ± 0.40, respectively. Peptides that correspond to the regions containing the respective point mutations in mutants L89G and S46Y are highlighted in red.

It has been shown previously that the fold stability of a protein influences the repertoire of presented peptides and that changing the stability can promote the presentation of hitherto not presented, so-called cryptic epitopes. To confirm our data from the *in vitro* degradation assay that displayed a very similar peptide profile for the different molecules, we again mapped the T cell epitopes of Phl p 6 in mice immunized with the WT protein or the mutants. As shown in Figure 11, mice immunized with the different Phl p 6 mutants elicited the same profile of T cell responses as the WT molecule. Only the group immunized with the destabilized mutant L89G also displayed a weak response against the region surrounding peptide 13, which may be due to the more relaxed state of L89G (*P* < 0.05 vs. S46Y, *P* = 0.057 vs. WT).

### Stabilized Mutant S46Y Shifts the Immune Polarization Toward Inflammatory Response Types

**Figure 12:**
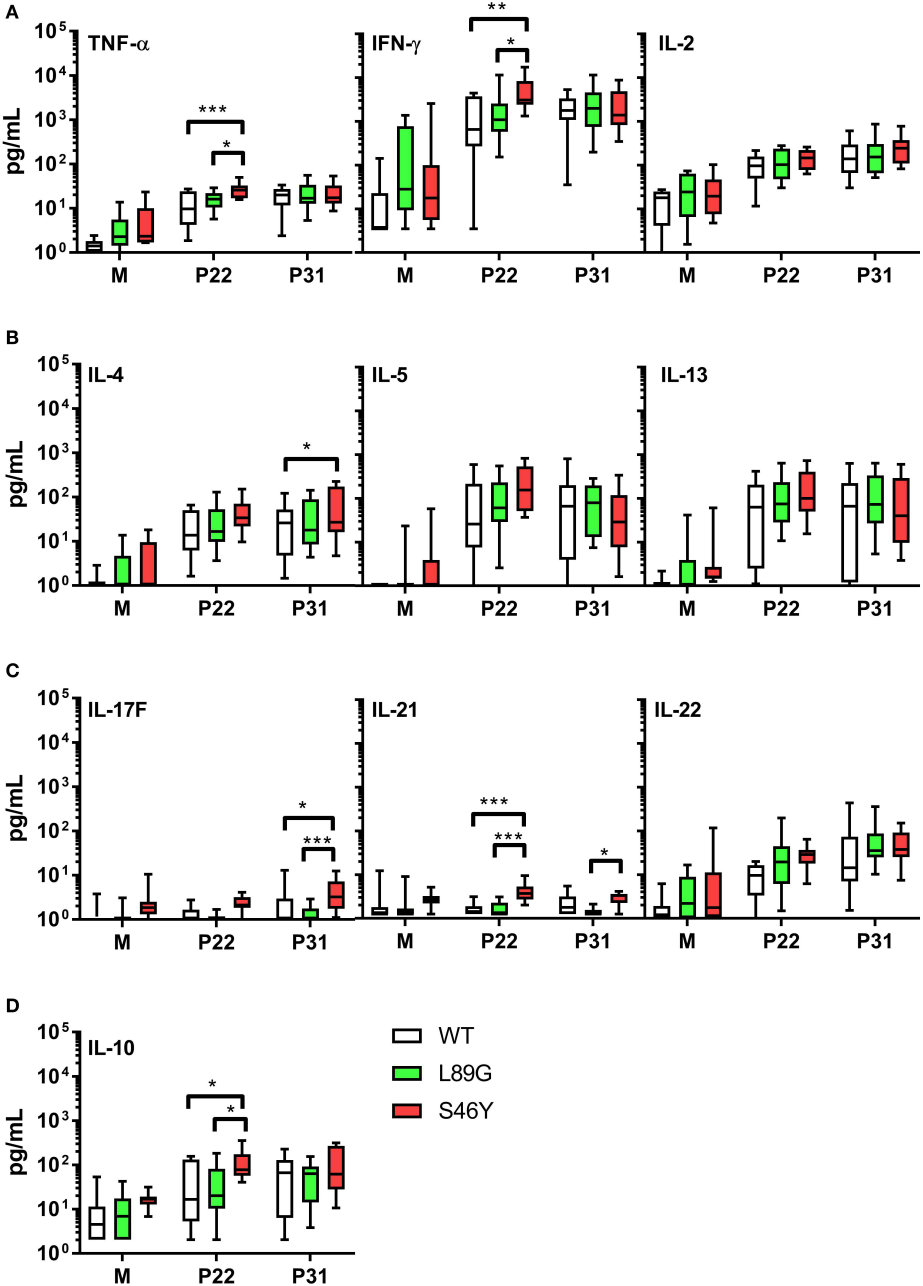
Immunization with stabilized mutant S46Y shifts the T cell response toward TH1/TH17. BALB/c mice were immunized with Phl p 6 wild-type (WT) protein, and mutants L89G and S46Y. Splenocytes from immunized mice were restimulated *in vitro* with peptide 65–79 (P22) or 92–106 (P31) or remained unstimulated (medium control, M) for 3 days. Cytokine concentrations in cell culture supernatants were measured by a flow cytometry based multiplex bead assay. Data are shown as box (25th−75th percentile) and whiskers (min to max) plots (*n* = 10). Statistical differences were assessed by two-way RM ANOVA followed by Tukey's *post-hoc* test. ^***^P < 0.001, ^**^P < 0.01, ^*^P < 0.05.

The amount of pMHCII complexes presented on the surface of APCs has been shown to influence the immune polarization of T cells *in vitro* and *in vivo*. Thus, we hypothesized that the different processing speed of mutant proteins with increased or reduced fold stability might also impact the immune polarization. Therefore, we stimulated splenocytes from immunized mice with the immunodominant peptides 65–79 or 92–106 and measured cytokine secretion in culture supernatants. Although stimulation with peptides 65–79 and 92–106 induced similar T cell proliferation in mice immunized with the wild-type protein and the mutants (Figure 11), the groups significantly differed in their cytokine profile. In line with the increased IgG2a levels indicating an enhanced TH1 immune response (Figure 9B), splenocytes derived from mice immunized with the stabilized mutant S46Y, secreted significantly higher levels of TH1 cytokines TNF-α and IFN-γ after stimulation with peptide 65–79 (Figure 12A).

Similarly, TH17 cytokines IL-17A (not shown), IL-17F, and IL-21 (Figure 12C) were increased. In contrast, TH2 cytokines IL-4, IL-5, and IL-13 were similar between all groups, with the exception of IL-4, which was slightly elevated in the S46Y group after stimulation with peptide 92–106 (Figure 12B). The regulatory cytokine IL-10 was also elevated in the S46Y group (Figure 12D), which might reflect a counter regulation due to the inflammatory response induced by S46Y.

## Discussion

Enormous efforts have been undertaken to understand and modulate activation and differentiation of TH cells into different effector cell types. The classical qualitative model postulates that MHCII-TCR interaction (signal 1) and co-stimulation via CD28 (signal 2) trigger activation and proliferation of naïve TH cells, whereas cytokines from extrinsic sources such as the APC itself or innate bystander cells largely determine immune polarization. In line with this, individual cytokines or combinations thereof have been identified to drive the development of specific T cell subsets, such as TH1 (IL-12), TH2 (IL-4), TH17 (TGF-β, IL-6, IL-21), Tfh (IL-21), TH9 (TGF-β, IL-4), iTreg (TGF-β, IL-2), and Tr1 (IL-10, IL-27) cells (53). However, accumulating evidence suggests that also the quantity and the quality of the TCR signaling induced by pMHCII stimulation plays a crucial role in T cell polarization. In support of this quantitative model, it has been shown that high doses of antigen increase the number of pMHCII complexes presented on the surface of the APC affecting TCR signal strength and/or duration. In summary, these studies suggest that weak TCR:pMHCII interactions favor TH2 or Tfh responses, whereas strong interactions induce TH1 responses (54). This model has important implications for our understanding why some proteins, such as allergens, seem to have intrinsic features that drive a certain type of immune polarization. We have previously suggested that protein fold stability represents such an intrinsic feature that has significant impact on the immunogenicity and allergenicity of proteins (55). Efficient antigen processing relies on antigen unfolding within the endolysosome. This process is mediated by endolysosomal acidification, and proteolytic cleavage of proteins by resident proteases, which preferentially degrade proteins in an unfolded state, facilitating access to the protein backbone (56). Additionally, proteins in a more relaxed state can serve as better substrates for binding to MHC II, which in turn can protect epitopes from further proteolytic degradation (57). However, there is very little *in vivo* data available focusing on the impact of fold stability on polarization of the immune response. Furthermore, studies investigating alterations of immune responses caused by modulated antigen stability have often been contradictory (19).

In our current study, we used an unbiased *in silico* approach to predict stability changes upon the introduction of single point mutations leaving known T cell epitopes and the overall structure unchanged. *In silico* predicted changes in free enthalpy (ΔΔG) correlated well with experimentally determined thermal stability as measured by CD spectroscopy and NMR. In the destabilized mutant L89G, a surface exposed leucine was replaced by a glycine, thereby removing the side chain at this position, giving adjacent amino acids additional flexibility, which was confirmed by aMD simulations. In mutant S46Y, a buried polar amino acid was replaced by a hydrophobic amino acid. Such replacements have been referred to as “hydrophobic core packing” and reported to have stabilizing effects on the protein structure by increasing the overall hydrophobicity within the protein core (22). Furthermore, the removal of cavities, by introduction of point mutations, has been shown to have stabilizing effects as well (58–60). During antigen processing, protein unfolding represents an indispensable step, as proteases require access to the backbone of the proteins for efficient cleavage (56). Following cellular uptake, antigens are exposed to a continuous drop in pH from mild acidic conditions in the early endolysosome down to pH 4.0 in terminal lysosomes. Fold stability, in particular resistance to pH-mediated denaturation, determines the extent and time-point of unfolding during lysosomal acidification. It has been shown that early endosomes (pH ~6) and terminal lysosomes (pH ~4.0–4.5) are poor in MHC II molecules whereas high quantities of MHC II are found in the late endosome (pH ~4.5–5.5), where the majority of MHC II loading takes place (61). We propose a model in which efficient antigen processing requires high fold stability in the early endosome, allowing the accumulation of intact antigen in the late endosome where it becomes sensitive to pH mediated unfolding, facilitating efficient proteolytic degradation (55). Thermal stability of Phl p 6 and its mutants correlated remarkably well with the speed of proteolytic digestion using endosomal extracts (Figures 6A,B) with the exception of N16M, which despite a considerable shift in melting point (+8.8°C) did not display enhanced protease resistance. This is in line with aMD simulations that indicated N16M to be extremely unstable, showing fast unfolding even at the nano-second timescale. Thus, aMD simulations may be a very useful tool to eliminate false positives from the MAESTRO *in silico* predictions. To assess the presentation of the immunodominant T cell epitope 92–106 in the context of proteins with different fold stability, we used BMDCs and a T cell hybridoma line specific for the respective pMHCII complex. IL-2 secretion by the hybridoma cells directly correlated with the amount of peptide loaded on BMDCs and thus represents a sensitive biological surrogate parameter to determine the amount of pMHCII complexes on the surface of APCs. We found that peptide 92–106 was much faster and more efficiently processed from L89G compared to the WT, whereas presentation was almost abrogated in the most rigid mutant S46Y. This is in line with the enhanced susceptibility to proteases of L89G observed in the degradome assay. We have previously found that immunodominant peptides from Bet v 1.0101 or tropomyosin (55, 62) are quickly degraded in the degradome assay, but nevertheless are effectively presented by BMDCs *in vitro*, supporting the concept of “bind first, cut later.” Kim et al. have shown that immunodominant epitopes are protected from further degradation by binding to MHC II, whereas epitopes sensitive to DM-mediated dissociation are destroyed by cathepsins (57, 63). Data on the protein dynamics from aMD simulations indicated that the introduction of glycin 89 enhanced the flexibility in the alpha helix (91–96) adjacent to the epitope (Figure 4) facilitating access to additional cleavage sites (Figures 6C,D) and/or binding to MHC II. Our finding that the efficacy of presentation for a given epitope is largely dependent on the fold stability of the protein is in line with both models.

Since we also wanted to investigate the impact of fold stability on the immunogenicity of the generated Phl p 6 derivatives in an adjuvant free setting *in vivo*, it was crucial to avoid any contaminations, which might modulate the antigen triggered immune response, in particular remaining LPS within the protein solutions. It was previously shown, that LPS concentrations of 0.2 ng/ml are sufficient to activate human monocytes and CD1c^+^ DCs *in vitro* (29). LPS from our recombinant proteins was therefore removed to <0.1 pg per μg protein, which corresponds to <2 pg/ml in the *in vitro* experiments and to <1 pg LPS per mouse for each immunization in the *in vivo* experiments. Therefore, we can exclude LPS induced effects in our experiments. Despite the much slower protein degradation *in vitro*, S46Y turned out to be significantly more immunogenic in terms of IgG2a induction, while IgG1 levels, serum and cell bound IgE remained unaltered (Figure 9). The observed increase in IgG2a antibodies is in line with a significantly altered cytokine response, showing a shift toward secretion of TH1 and TH17 cytokines after stimulation with the immunodominant peptides (Figure 12). Various other studies have investigated the effect of fold stability on the immunogenicity of proteins and the subsequent T cell polarization with divergent results. Some reported a higher immunogenicity of stabilized proteins with respect to antibody titers (64, 65). Others have found exactly the opposite, namely a reduced immunogenicity of stabilized variants (66, 67) and an increased immune response due to an elevated peptide presentation in destabilized proteins (56). Further, the immunogenicity is reduced upon protein denaturation (68) and increased protease susceptibility (23). With regards to T cell polarization, a stabilized form of Bet v 1, including a short Mal d 1 sequence, elicited an increased TH1 response compared to the wild-type Bet v 1 (65). In contrast, we found an increased TH2 response for Bet v 1 containing stabilizing point mutations in comparison to WT Bet v 1 (55). For hen egg lysozyme (HEL), induction of an increased amount of IL-4 could be detected for the destabilized variant, suggesting an increased TH2 response. However, the amount of IFN-γ was also elevated after immunization with the destabilized HEL and not detectable for the stabilized form, indicating an overall higher immune response (69). We postulate a model (19) where proteins with high protease susceptibility are rapidly degraded in the early endosome, resulting in low numbers of pMHCII complexes to be loaded in the late endosomal MHC class II compartment (MIIC). Only proteins with sufficient stability survive long enough to reach the MIIC where they are processed and loaded on MHC II. However, hyperstable proteins that fail to unfold in the MIIC cannot be processed at all and are thus not immunogenic (55). Consequently, an optimal stability will generate a high density of pMHCII, which is important for TCR signaling and induction of a TH1 response (9, 70). Deviation from this optimum—in both directions—can result in TH2 polarization. This would explain, why in different studies, depending on the initial stability of the used protein, additional stabilization can lead to divergent results.

Mutations in the native protein (71, 72), very high antigen concentrations, or unusual protein conformations (73, 74) have been shown to induce presentation of so-called cryptic epitopes that are usually not presented. In this context, we investigated whether changes in fold-stability, which resulted in substantial changes in the processing kinetics *in vitro*, would also change the epitope usage *in vivo*. However, as shown in Figure 11, the epitope usage between WT, L89G, and S46Y remained largely unchanged, with the exception of the region around peptide 13, which was only recognized by T cells from mice immunized with L89G, indicating that the more relaxed conformation of L89G resulted indeed in the presentation of a cryptic epitope.

In summary, MAESTRO was very efficient in detecting single point mutations that increase or reduce fold-stability, at least for small, alpha-helical proteins such as Bet v 1 (55) or Phl p 6, and aMD simulations could be successfully used to eliminate false positives. Thermal stability correlated well with susceptibility to protease resistance and presentation of pMHCII on the surface of BMDCs *in vitro*. Surprisingly, more efficient processing in these *in vitro* systems did not correlate with enhanced immunogenicity and TH1 polarization *in vivo*. On the contrary, the more stable mutant S46Y turned out to be more immunogenic and TH1 polarizing than the destabilized mutant L89G. Potentially, in the *in vivo* setting, either the enhanced protease resistance of S46Y provides the molecule with the advantage of longer extracellular survival (depot effect), or the *in vivo* situation requires slower antigen processing compared to the *in vitro* assays. This applies especially for intradermal injections (as used in this study) where skin dendritic cells take up the antigen and migrate to skin draining lymph nodes during a time-period of 24–48 h before they encounter their matching T cells. Additional experiments are necessary to test this hypothesis. Taken together, we have shown that *in silico* evaluation of fold stability modulated proteins has the potential to optimize and polarize immune responses, which opens the door to more efficient design of molecular vaccines.

## Data Availability Statement

The datasets generated for this study can be found in the Protein Data Bank under the entry code 6TRK.

## Ethics Statement

The animal study was reviewed and approved by Austrian Ministry of Education, Science and Research (permit no. BMWF-66.012/0013-WF/V/3b/2017].

## Author Contributions

PW, SSt, SSc, IJ, and RW expressed, purified, and characterized recombinant proteins and performed *in vitro* degradation and presentation assays and *in vivo* experiments. LS, CB, and CH performed mass spectrometry and analyzed the respective data. WS and JB performed X-ray crystallography and analyzed the structures. FH, AK, and KL developed aMD algorithms and performed the respective analyses. VD and MT expressed proteins for NMR and performed NMR spectroscopy. JL and PL designed the MAESTRO algorithm, modeled the Phl p 6 structure for analysis and performed MAESTRO analyses. SM and JH-H performed masked LPS assays and analyzed the respective data. SSc and RW designed the study and wrote the manuscript. All authors reviewed, revised, and approved the final manuscript.

## Conflict of Interest

The authors declare that the research was conducted in the absence of any commercial or financial relationships that could be construed as a potential conflict of interest.
