# Supplementary material for: In silico Design of Phl p 6 Variants With Altered Fold-Stability Significantly Impacts Antigen Processing, Immunogenicity and Immune Polarization
Source: Front Immunol. 2020 Aug 18;11:1824. doi: 10.3389/fimmu.2020.01824 (PMC7461793; doi:10.3389/fimmu.2020.01824)
Supplement: Supplementary file 1 [file Data_Sheet_1.PDF]

**Supplementary Table 1. Crystallographic data and refinement statistics.**

| Structure | S46Y | S46Y, Zn peak | S46Y, Zn remote |
|---|---|---|---|
| **Data collection** | | | |
| **Wavelength (Å)** | 0.9763 | 1.2815 | 1.2848 |
| **Unit cell parameters** | | | |
| ***a,b,c (Å)*** | 47.83, 73.51, 76.12 | 47.49, 73.13, 75.78 | 47.65, 73.29, 75.91 |
| ***α,β,γ (degrees)*** | $\alpha=\beta=\gamma=90$ | $\alpha=\beta=\gamma=90$ | $\alpha=\beta=\gamma=90$ |
| **Space group** | $P22_12_1$ | $P22_12_1$ | $P22_12_1$ |
| **Solvent content (%)** | 56.64 | 56.64 | 56.64 |
| **Protein chains in AU** | 2 | 2 | 2 |
| **Resolution range (Å)** | 39.94-1.60 | 47.79-1.61 | 47.65-1.61 |
| **Highest resolution shell (Å)** | 1.69-1.60 | 1.69-1.61 | 1.70-1.61 |
| **Unique reflections** | 34555 (5013) | 35203 (5075) | 35198 (5072) |
| **Redundancy** | 3.5 (3.1) | 7.2 (7.3) | 7.3 (7.4) |
| **Completeness (%)** | 96.9 (97.5) | 99.9 (100) | 99.9 (100) |
| ***$R_{merge}$** | 0.070 (0.439) | 0.083 (0.794) | 0.064 (0.424) |
| **$R_{meas}$** | 0.082 (0.523) | 0.104 (0.926) | 0.074 (0.493) |
| **Average $I/\sigma(I)$** | 9.3 (2.3) | 10.9 (2.2) | 14.3 (3.7) |
| **Refinement** | | | |
| **$R_{work}$ (%)** | 15.52 | | |
| **$R_{free}$ (%)** | 18.27 | | |
| **Mean *B* value ($Å^2$)** | 29 | | |
| **B from Wilson plot ($Å^2$)** | 16.9 | | |
| **RMSD bond length (Å)** | 0.007 | | |
| **RMSD bond angles (°)** | 0.859 | | |
| **No. of amino acid residues** | 108 | | |
| **No. of water molecules** | 303 | | |
| **No. of metal ions** | 4 | | |
| **Ramachandran plot** | | | |
| **Most favored regions (%)** | 99.55 | | |
| **Allowed regions (%)** | 0.45 | | |

Values of the highest resolution shell are given in parentheses.

$*R_{merge} = \Sigma_h\Sigma_l |I_{hl} - \langle I_h\rangle| / \Sigma_h\Sigma_l |\langle I_h\rangle|$
